# Supplementary material for: Modeling of longitudinal immune profiles reveals distinct immunogenic signatures following five COVID-19 vaccinations among people living with HIV
Source: Patterns (N Y). 2026 Mar 4;7(3):101474. doi: 10.1016/j.patter.2025.101474 (PMC13100683; doi:10.1016/j.patter.2025.101474)
Supplement: Document S1. Figures S1–S12 and Tables S1–S3 [file mmc1.pdf]

Patterns, Volume *7*

Supplemental information

Modeling of longitudinal immune profiles reveals distinct immunogenic signatures following five COVID-19 vaccinations among people living with HIV

Chapin S. Korosec, Jessica M. Conway, Vitaliy A. Matveev, Mario Ostrowski, Jane M. Heffernan, and Mohammad Sajjad Ghaemi

Supplementary Material: Modelling of longitudinal immune profiles reveals distinct immunogenic signatures following five COVID-19 vaccinations among people with HIV

Chapin S. Korosec[1,2,3], Jessica M. Conway[4], Vitaliy A. Matveev[5], Mario Ostrowski[5,6,7], Jane M. Heffernan[2,3], Mohammad Sajjad Ghaemi[8]

[1]Department of Mathematics and Statistics, University of Guelph, Guelph, ON, Canada.[2]Modelling Infection and Immunity Lab, Mathematics and Statistics, York University, Toronto, ON, Canada.[3]Centre for Disease Modelling, Mathematics and Statistics, York University, Toronto, ON, Canada.[4]Department of Mathematics, Pennsylvania State University, University Park, Pennsylvania, United States of America.[5]Department of Medicine, University of Toronto, Toronto, ON, Canada.[6]Department of Immunology, University of Toronto, Toronto, ON, Canada.[7]Keenan Research Centre for Biomedical Science, St. Michael's Hospital, Unity Health, Toronto, ON, Canada.[8]Digital Technologies Research Centre, National Research Council Canada, Toronto, ON, Canada.

*Corresponding authors: chapinSkorosec@gmail.com, jmheffer@yorku.ca, mohammadsajjad.ghaemi@nrc-cnrc.gc.ca

Contents

S1 ML benchmark analysis **3**

S2 Additional detail on biomarker descriptions **4**

S3 PWH viral load metadata **5**

S4 RF classification probabilities **8**

S4.1 Synthetic Data Analysis 13

S5 Imputation analysis **16**

S6 UMAP to compliment tSNE **19**

S1 ML benchmark analysis

**Method**	**Accuracy**	**ROC AUC**	**PR AUC**	**Precision**	**Recall**	F_1
Logistic Regression	0.83	0.85	0.92	0.95	0.82	0.88
LDA	0.82	0.87	0.94	0.94	0.81	0.87
SVM (linear)	0.95	0.99	1	0.96	0.98	0.97
SVM (RBF)	0.92	0.99	1	0.92	0.98	0.95
Greedy Forward	0.87	0.93	0.98	0.95	0.88	0.91
Greedy Backward	0.82	0.85	0.92	0.93	0.82	0.87
**Random Forest**	0.94	0.99	1	0.94	1	0.97

Table S1: **Benchmark of classification methods under identical cross-validation.** All models were evaluated with stratified 5-fold *outer* CV; where applicable, hyperparameters were selected by stratified 5-fold *inner* CV (training fold only). Values are outer-fold means (rounded to two significant figures). Positive class probabilities were used to compute ROC AUC and PR AUC; thresholded metrics used a 0.5 cutoff.

S2 Additional detail on biomarker descriptions

**Immunological Feature (63 total)**	**Brief Description**
IgG Serum Spike	Systemic IgG antibodies in blood targeting the SARS-CoV-2 spike protein; indicates systemic humoral immunity.
IgG Serum RBD	IgG in blood targeting the receptor-binding domain (RBD) of spike; involved in virus neutralization.
IgG Saliva Spike	Mucosal IgG in saliva targeting spike; reflects mucosal immune response.
IgG Saliva RBD	Mucosal IgG in saliva targeting the RBD; may correlate with protection at mucosal surfaces.
IgA Saliva Spike	Secretory IgA in saliva targeting spike; key for mucosal immunity, especially prevention of initial infection.
IgA Saliva RBD	Secretory IgA in saliva targeting the RBD; key for mucosal immunity, especially prevention of initial infection.
IFNγ (Interferon γ)	Cytokine produced by activated T cells (especially Th1); critical for antiviral immunity and macrophage activation.
IL-2 (Interleukin-2)	Cytokine produced by activated T cells that promotes T-cell proliferation and survival; important for immune memory.
Dual (IL-2 + IFNγ)	T cells simultaneously producing IL-2 and IFNγ; polyfunctional responses associated with robust immunity.
Neutralization	Functional antibody assay measuring the ability of antibodies to prevent viral infection of host cells.
ACE2 Displacement	Surrogate for neutralization; measures the ability of antibodies to block viral RBD binding to the ACE2 receptor.

Table S2: Overview of the immunological feature families used in this study. Individual measurements (63 total) fall into the categories listed; detailed assay descriptions and per-feature metadata are provided in Methods/Supplement.

S3 PWH viral load metadata

Table S3: Patient metadata summary.

Number	Patient ID	HIV diagnosis	Years VL suppressed
24	CIRC 0054	1988	LTNP
25	OM 215	1993	28
26	OM 5085	1985	20
27	OM 5076	1989	21
28	CIRC 0022	1995	23
29	OM 5056	1987	24
30	CIRC 0116	2000	19
31	OM 5030	1985	20
32	CIRC 0050	1989	23
33	OM 5135	1988	17
34	OM 5208	1989	17
35	CIRC 0120	1989	16
36	CIRC 0313	1984	20
37	OM 5051	1990	23
38	CIRC 0041	1999	22
39	OM 5202	2008	12
40	CIRC 0280	1992	11
41	OM 5055	2002	25
42	OM 5013	1989	25
43	CIRC 0066	1989	24
44	OM 5128	2002	17
45	OM 5130	1990	16

Continued on next page

Continued from previous page

Number	Patient ID	HIV diagnosis	Years VL suppressed
46	OM 5265	2011	10
47	OM 5232	2000	21
48	OM 5368	2015	5
49	OM 5407	1992	7
50	OM 5248	1998	20
51	OM 5365	1990	18
52	OM 5200	1990	24
53	OM 5225	1999	21
54	CIRC 0196	2007	8
55	CIRC 0281	1997	13
56	CIRC 0146	1990	15
57	CIRC 0113	1993	19
58	CIRC 0188	1996	20
59	CIRC 0323	1990	19
60	OM 5213	1993	20
61	CIRC 0273	2000	16
62	CIRC 0302	2000	17
63	CIRC 0164	2006	11
64	OM 5122	2007	13
65	OM 5409	2007	12
66	CIRC 0028	1991	30
67	CIRC 0324	1987	16
68	OM 5400	1989	15
69	OM 5005	1987	15
70	CIRC 0216	2000	17

Continued on next page

Continued from previous page

Number	Patient ID	HIV diagnosis	Years VL suppressed
71	OM 5016	1992	14
72	CIRC 0322	1990	14
73	CIRC 0266	1988	16
74	CIRC 0060	1994	24
75	OM 5168	1985	20
76	OM 5156	1985	14
77	CIRC 0270	1985	22
78	CIRC 0274	2000	20
79	OM 5131	1982	15
80	CIRC 0319	1990	30
81	OM 5244	1986	20
82	CIRC 0058	1993	23
83	OM 5226	2005	16
84	CIRC 0174	1994	20
85	CIRC 0036	1991	30
86	CIRC 0308	1988	19
87	OM 5094	1985	NA
88	OM 5019	2005	NA
89	OM 5004	1981	NA
90	CIRC 0106	1990	NA
91	OM 5211	2000	NA

S4 RF classification probabilities

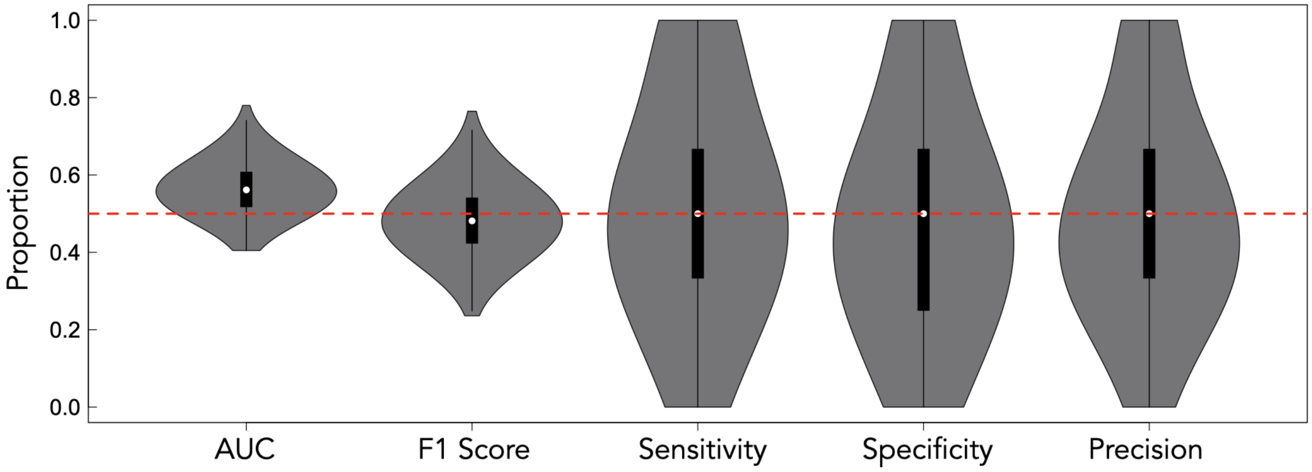

Figure S1: **Model performance with randomized labels using all 63 features.** RF was carried out similarly to as described in the methods section with K-fold CV, however, the labels were randomized. Provided are the AUC, F1 Score, Sensitivity, Specificity, and Precision, respectively, which can be compared to the true model performance metrics provided in Fig 1E. A red dashed line at 0.5 is shown for comparison

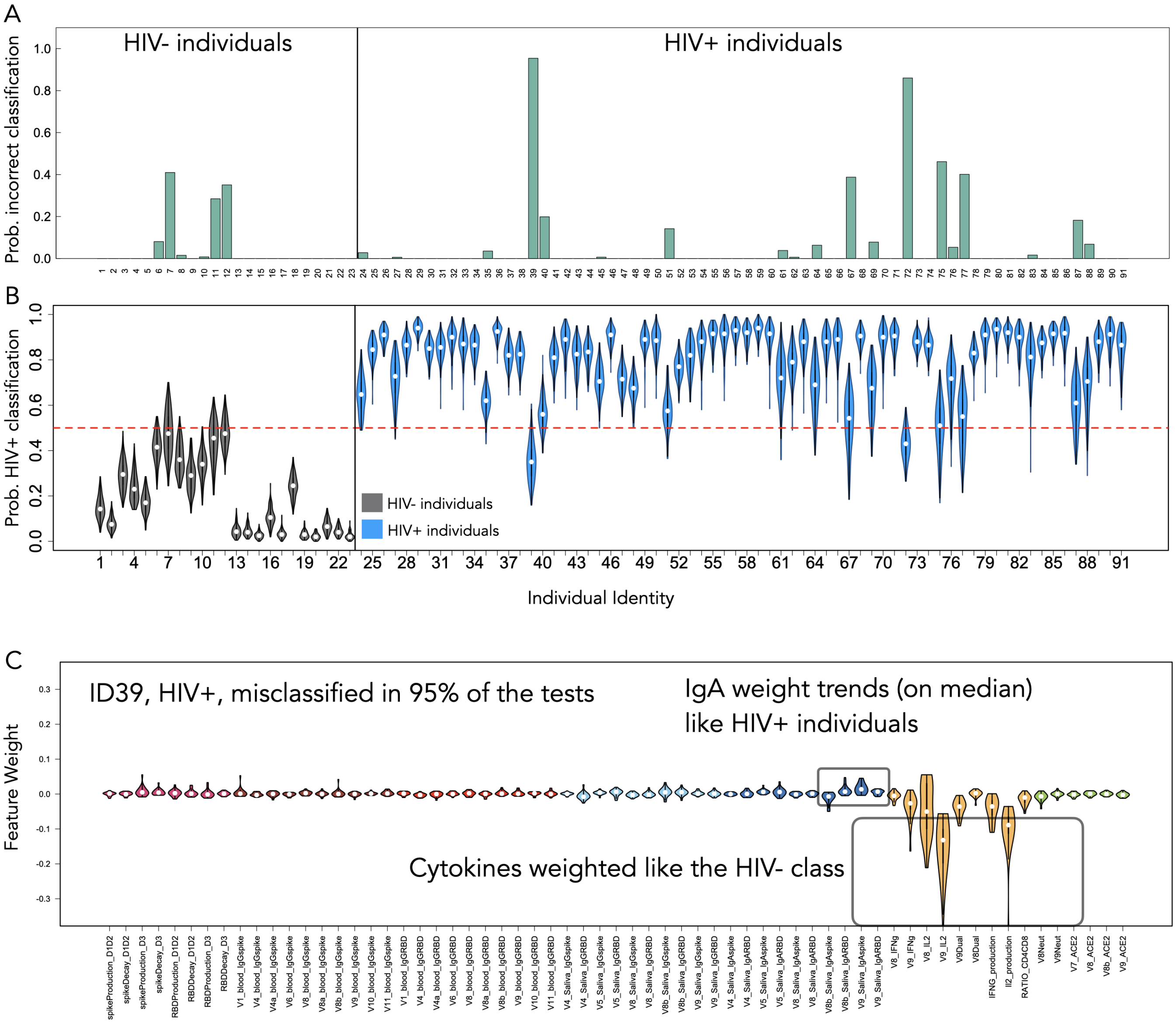

Figure S2: **Classification probabilities across all folds for HIV- and HIV+ individuals and misclassified HIV+ feature weight signature.** A) Probability that the RF model will incorrectly classify the individual, for all individuals in the full-feature RF models. B) Violin plots for the distributions of HIV+ classifications for all individuals. C) Example feature weight distributions for ID39, who tends to be misclassified as HIV- when they are known to be HIV+.

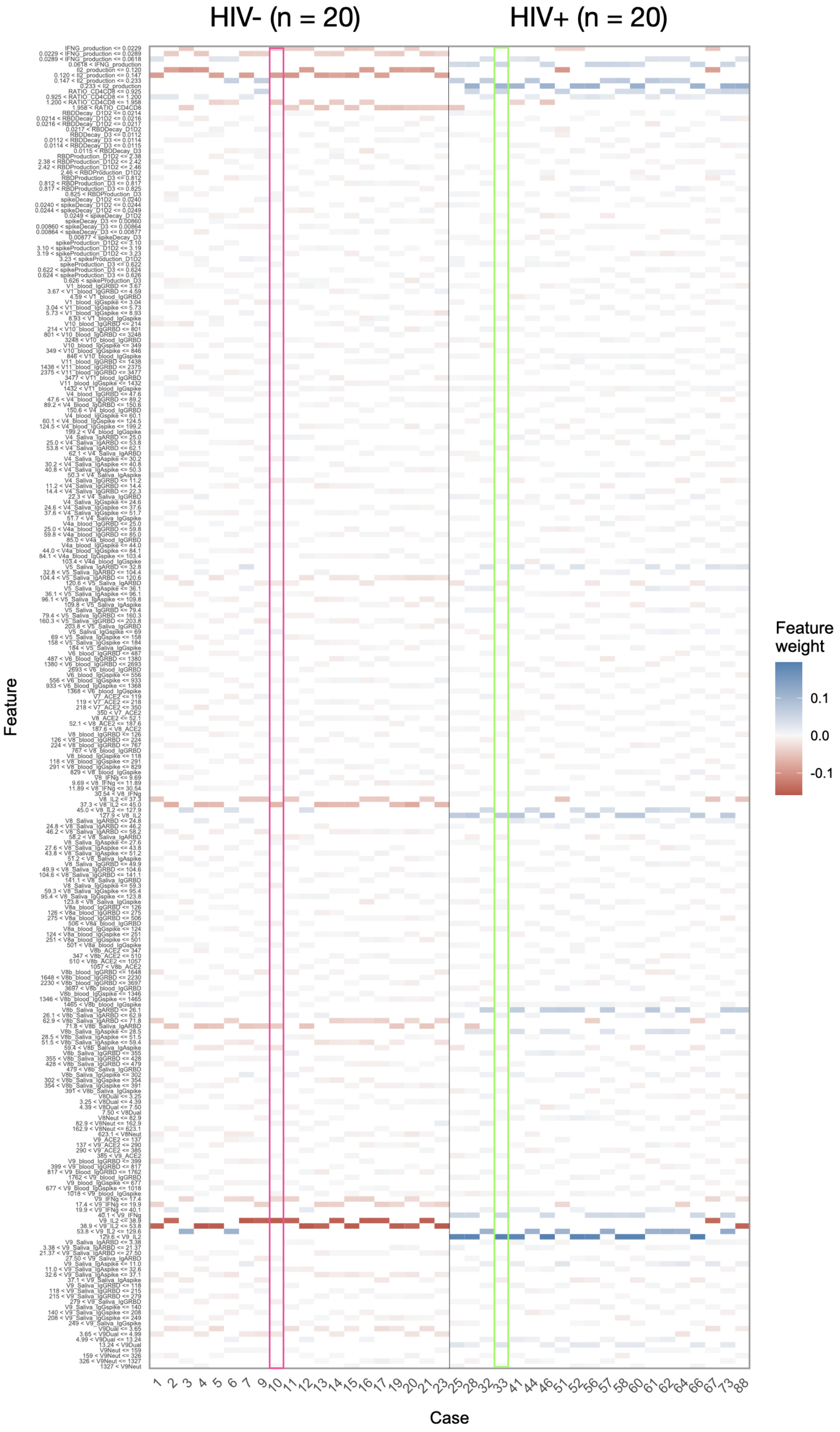

Figure S3: **RF training landscape example.** An example of the RF training landscape for a single RF model, IDs 10 and 33 are randomly highlighted, with their corresponding individual feature distributions shown in Fig. S4. This RF model produces an AUC of 1.0.

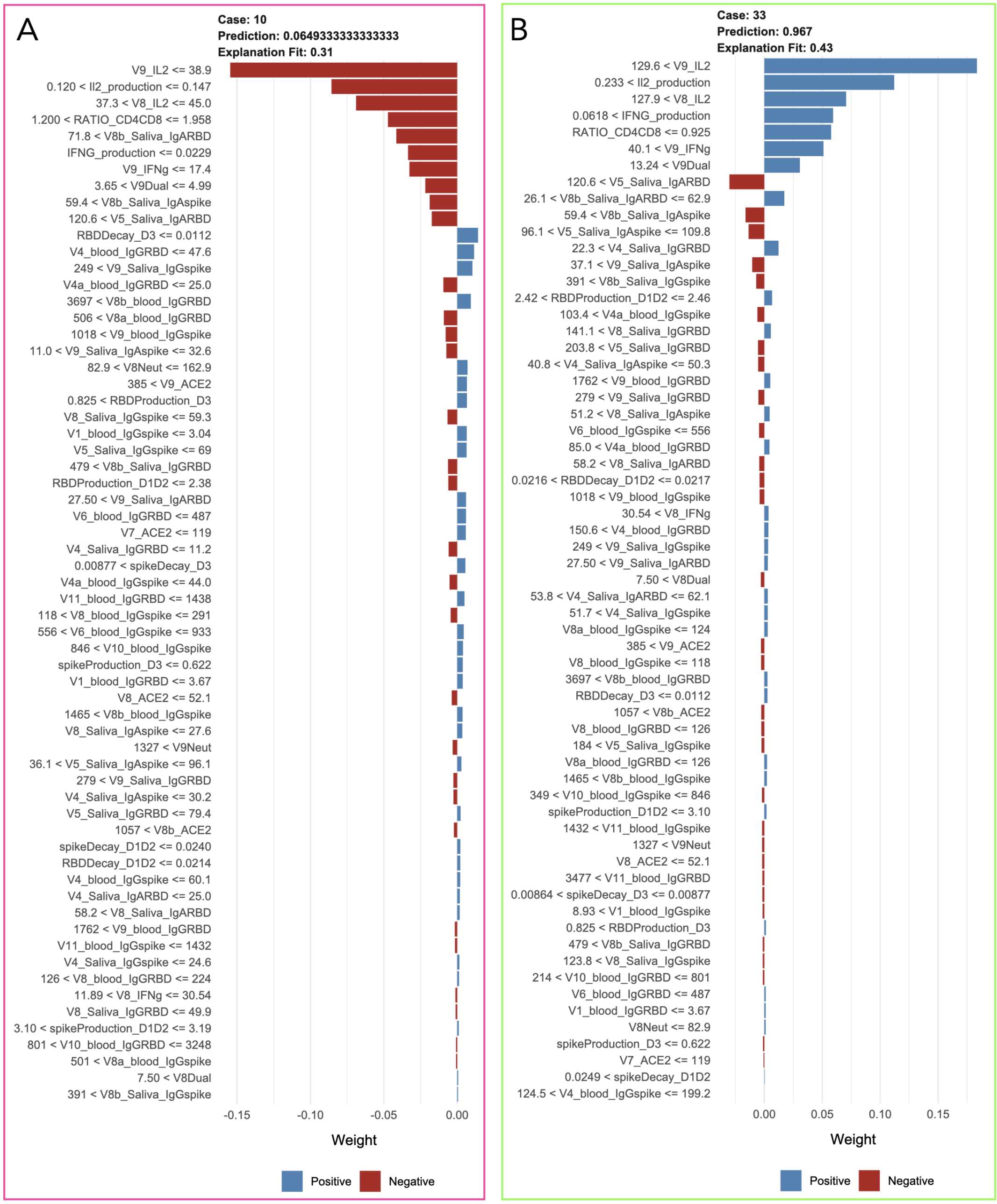

Figure S4: **Individual feature weights.** Individual feature weights for IDs 10 and ID 33 for a single RF model trained on all 64 features from the model landscape shown in Fig. S3.

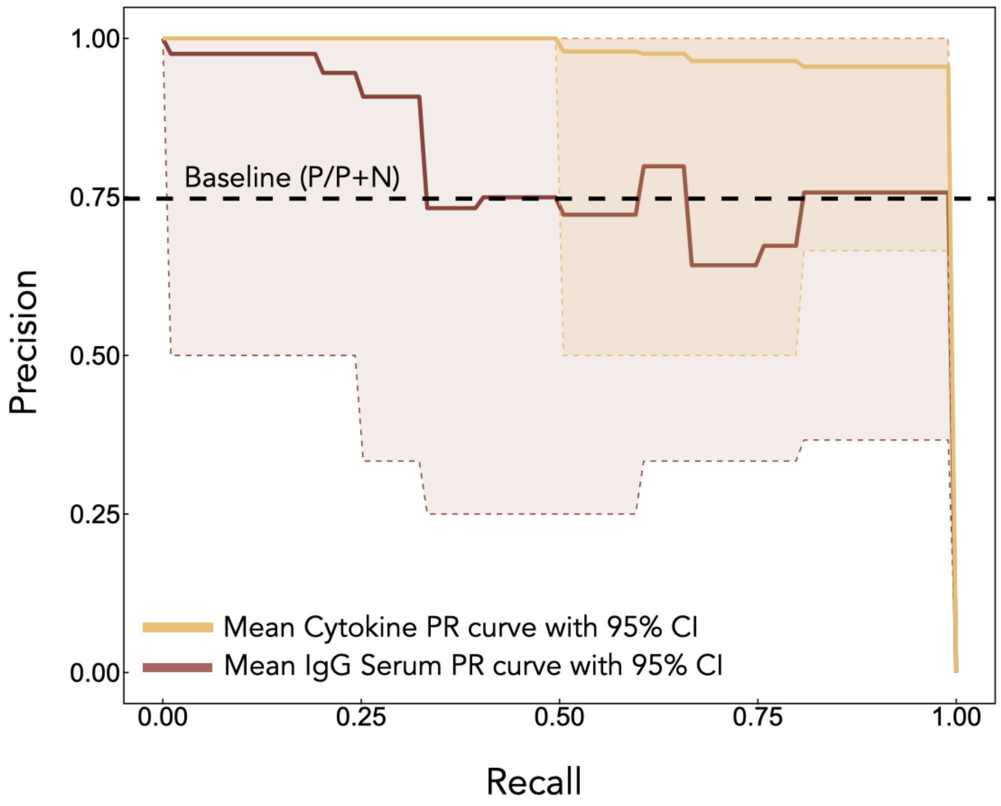

Figure S5: **Mean PR curves for RF models trained on just the cytokine features (yellow) and serum data (red).** Shaded regions represent 95% confidence intervals. The baseline (dashed line) is calculated by computing the ratio $P/(P+N)$ which is the ratio of positives and negatives in the full data set (not the downsampled balanced ratio of 1:1 used for training, but the representative ratio used for testing). In accordance with intuition gained from the mean ROC curves (Fig. 3E), the cytokine features result in a near-perfect classifier while serum features result in approximately baseline (random) performance.

S4.1 Synthetic Data Analysis

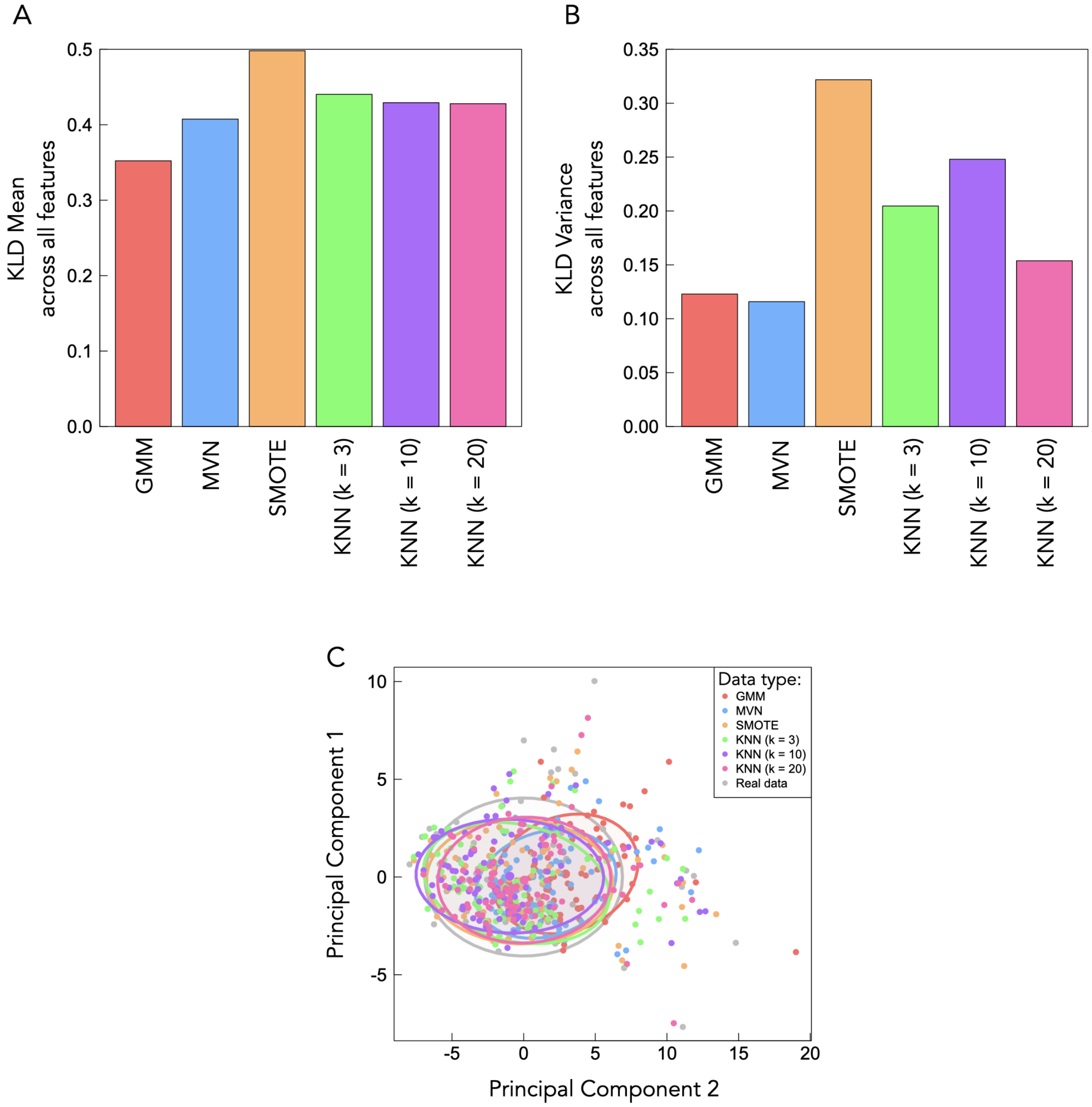

Figure S6: **Synthetic data KLD and PCA analysis.** A) Mean KLD is computed across all features between each respective synthetic data set and the real data. B) The variance in KLD is computed across all features between each respective synthetic data set and the real data. C) PCA analysis is displayed whereby the principal components from the real data are projected onto each respective principal components of the synthetic data. Ellipses are first standard deviation. Here, all synthetic data approaches are reasonably approximate the actual data and no meaningful cluster separation is found.

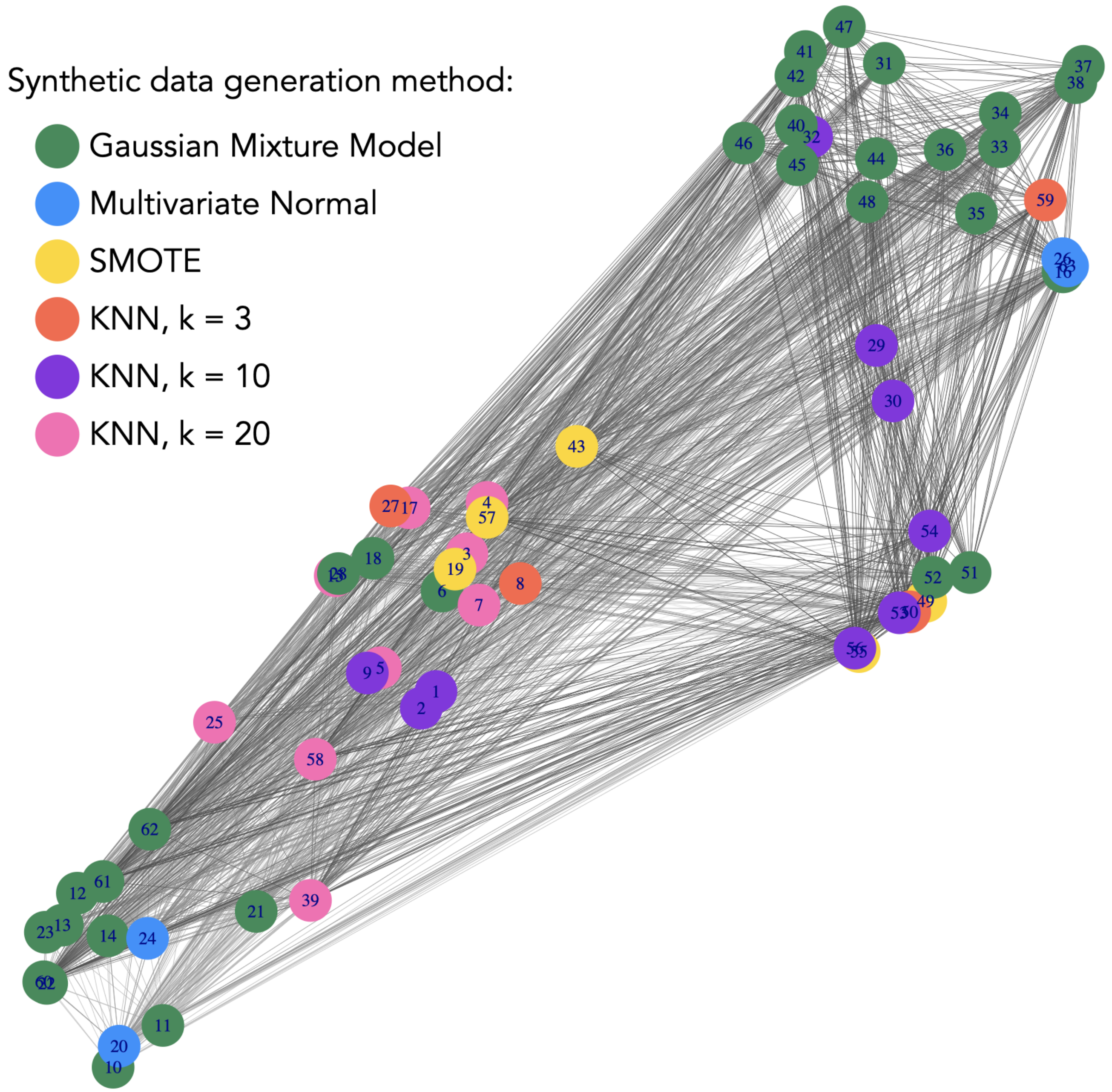

Figure S7: t-SNE plot shown with the same layout as in the maintext, however, colours here correspond to the synthetic data feature method that minimized the KLD for that specific feature.

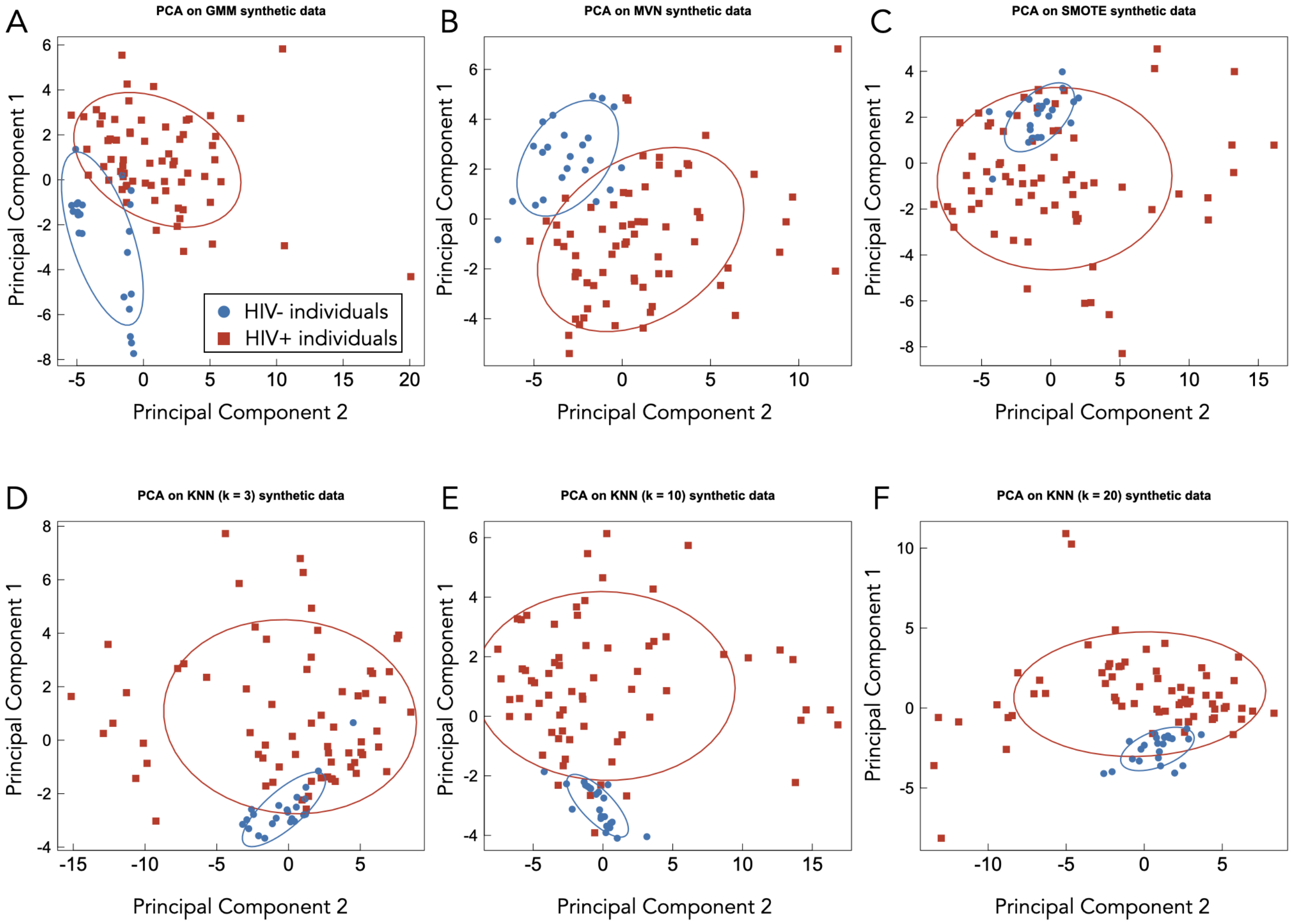

Figure S8: **PCA on synthetically generated data**. Panels A-F display the results of PCA performed on the synthetically generated data. Panels are ordered by GMM, MVN, SMOTE, KNN (k = 3), KNN (k = 10), and KNN (k = 20), for panels A, B, C, D, E, and F, respectively. SMOTE, despite having the highest mean KLD, is the only technique whereby the control class subclusters within the first standard deviation ellipse of the target class, which is similar structural behaviour observed by the actual data (Fig.1C). SMOTE is the only supervised method used to generate synthetic data in this work.

S5 Imputation analysis

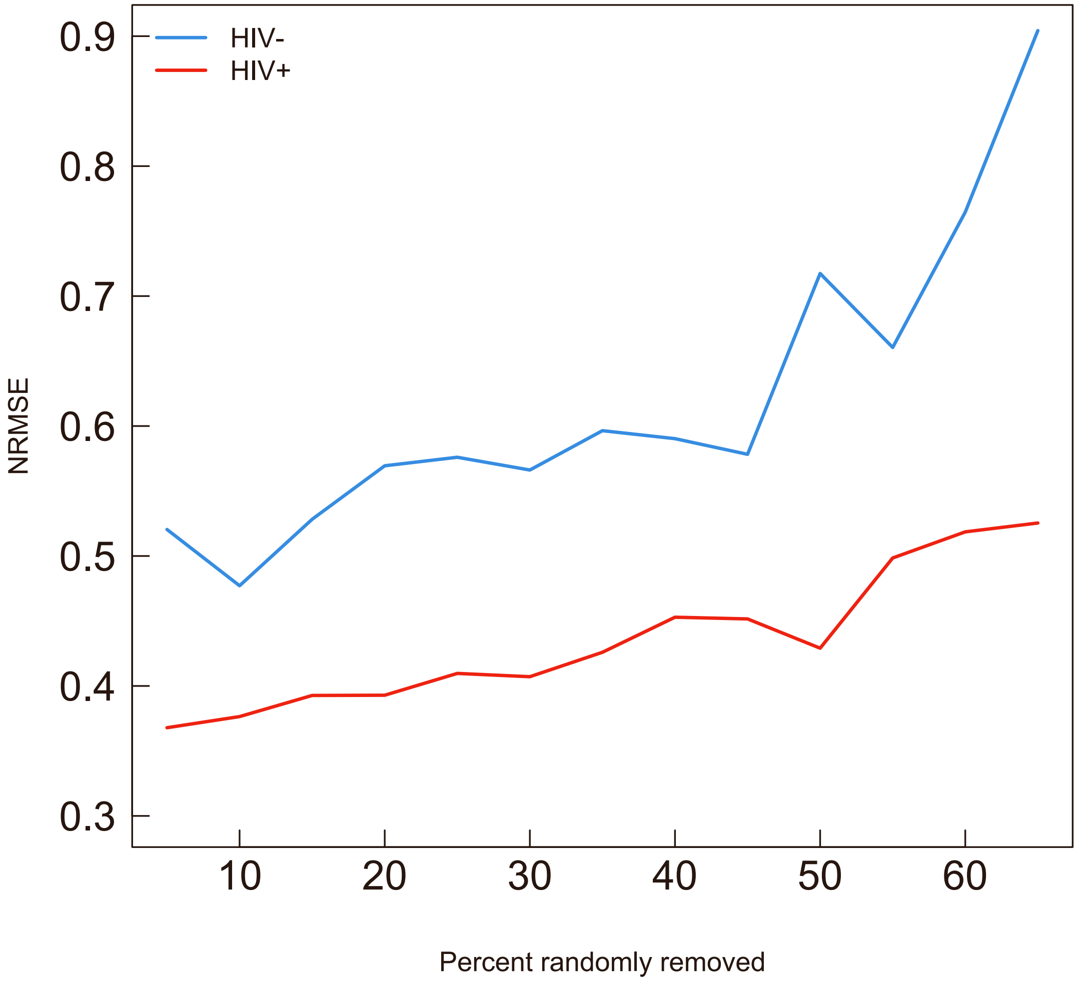

Figure S9: **Normalized Root Mean Squared Error (NRMSE) of imputed features as a function of increasing missingness.** NRMSE was computed separately for HIV-negative (blue) and HIV-positive (red) groups, with each point representing the average NRMSE across all features at a given level of missing data. As expected, NRMSE increases gradually as the proportion of missing data increases, reflecting the increasing uncertainty in imputed values. However, the shallow slope suggests that the imputation method remains robust across varying levels of missingness, preserving data integrity even at higher rates of removal.

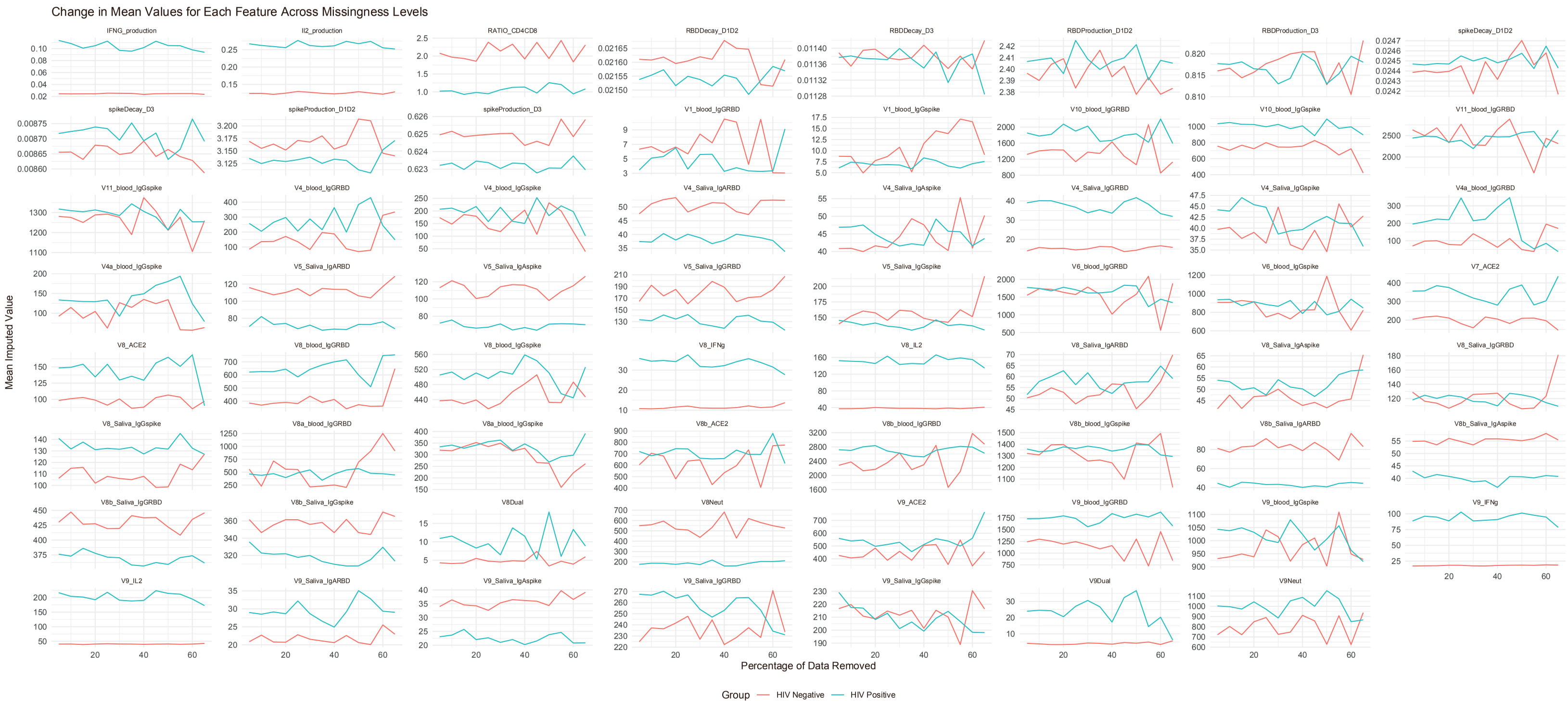

Figure S10: **Mean values of the imputed features remains stable across increasing levels of missingness.** Each panel represents a single feature, with the x-axis indicating the percentage of data removed prior to imputation, and the y-axis representing the mean of the imputed values. Imputation was performed separately for HIV-negative (blue) and HIV-positive (red) groups as described in the methods section. The relative stability of these metrics suggests that the imputation approach preserves the underlying data structure, even as missingness increases.

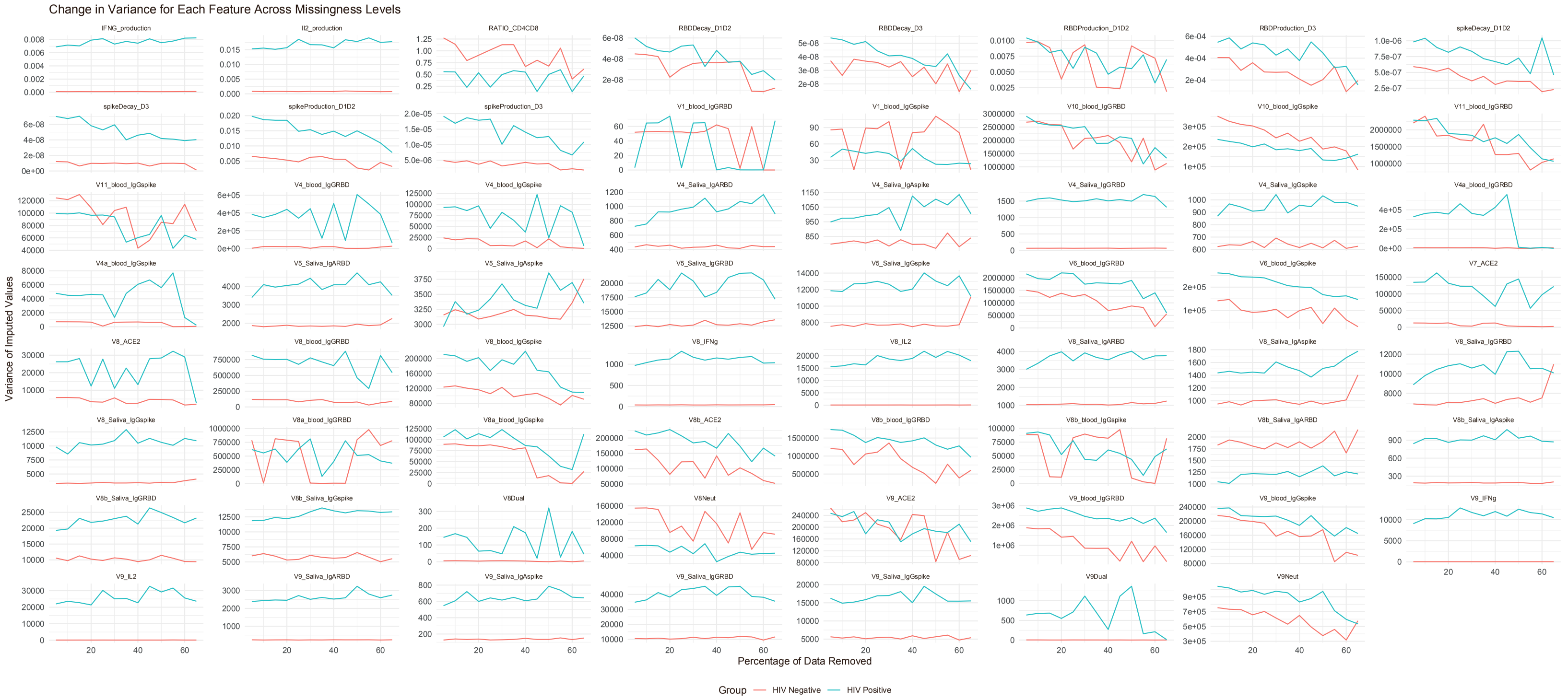

Figure S11: **Variance of the imputed features remains stable across increasing levels of missingness**. Each panel represents a single feature, with the x-axis indicating the percentage of data removed prior to imputation, and the y-axis representing the mean of the imputed values. Imputation was performed separately for HIV-negative (blue) and HIV-positive (red) groups as described in the methods section. The relative stability of these metrics suggests that the imputation approach preserves the underlying data structure, even as missingness increases.

S6 UMAP to compliment tSNE

As validation to confirm the stability and biological relevance of the tSNE clusters presented in Figure 2, here we look at feature clustering behvaiour using UMAP. We use the umap function in the R library uwot and set the effective neighbors to the same as the perplexity value used in the tSNE analysis.

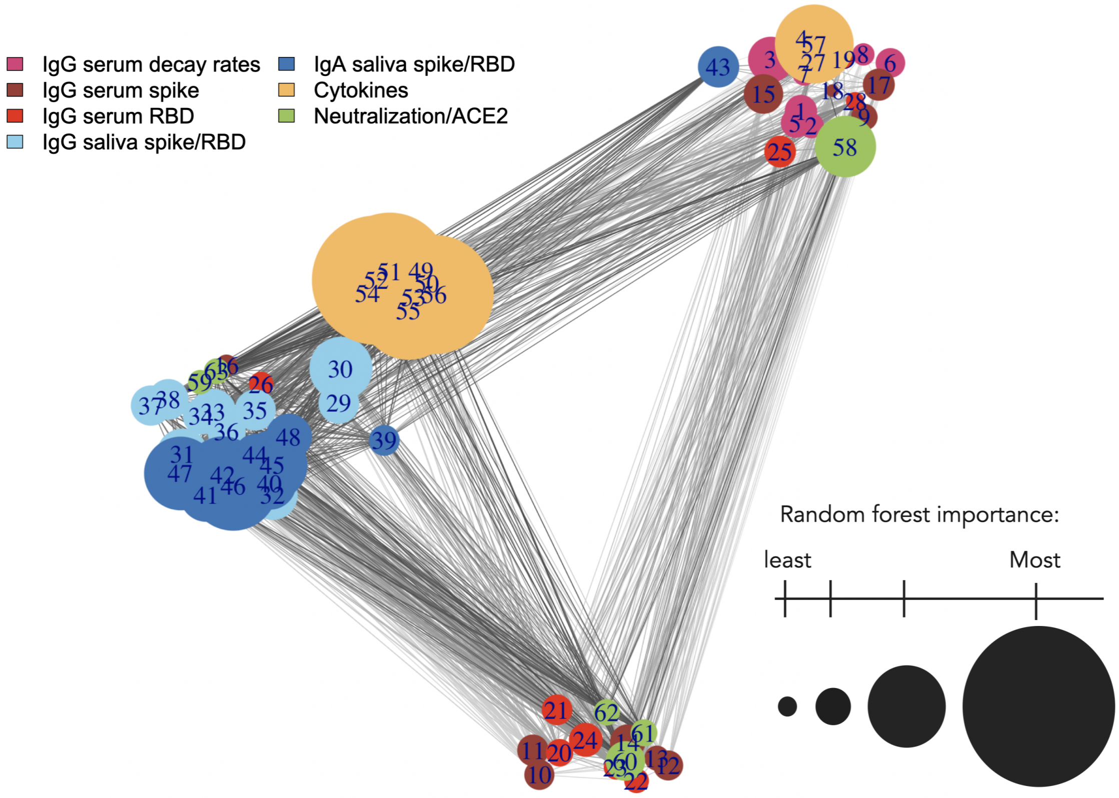

Figure S12: **UMAP of the feature correlation network.** UMAP embedding of the feature–feature correlation network. Nodes (1–63) are immune features; edges connect pairs with Spearman associations across the 91 participants with $p < 0.05$. The layout uses UMAP with $n_{\text{neighbors}} \approx (63-1)/3 = 20$, `min_dist`$= 0.1$, and Euclidean metric. Node colours denote assay families (magenta: IgG serum decay rates; red: IgG serum spike; maroon: IgG serum RBD; light blue: IgG saliva spike/RBD; dark blue: IgA saliva spike/RBD; orange: Cytokines; green: Neutralization/ACE2). Node size is proportional to random-forest feature importance.
