# Supplementary material for: Modeling of longitudinal immune profiles reveals distinct immunogenic signatures following five COVID-19 vaccinations among people living with HIV
Source: Patterns (N Y). 2026 Mar 4;7(3):101474. doi: 10.1016/j.patter.2025.101474 (PMC13100683; doi:10.1016/j.patter.2025.101474)
Supplement: Document S2. Article plus supplemental information [file mmc2.pdf]

Patterns

Modeling of longitudinal immune profiles reveals distinct immunogenic signatures following five COVID-19 vaccinations among people living with HIV

Graphical abstract

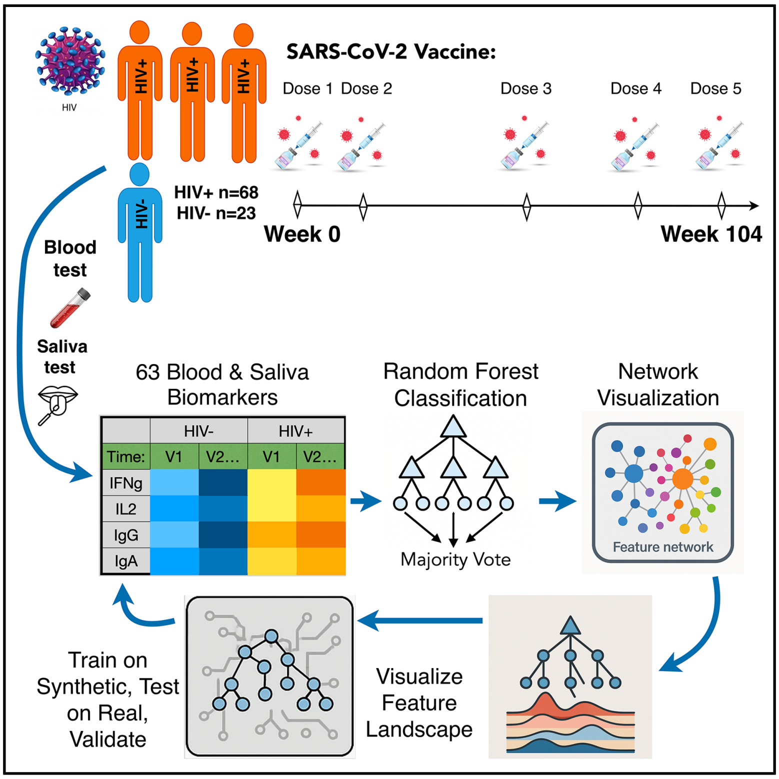

Highlights

- t-SNE reveals clustered immune features distinguishing humoral and cellular immunogenicity
- Interpretability maps show feature-weight patterns exposing atypical HIV immune responses
- Synthetic data enable accurate random-forest classification of real vaccine immunity
- Ablation analyses expose minimal immune feature sets driving robust classification

Authors

Chapin S. Korosec, Jessica M. Conway, Vitaliy A. Matveev, Mario Ostrowski, Jane M. Heffernan, Mohammad Sajjad Ghaemi

Correspondence

chapinskorosec@gmail.com (C.S.K.), MohammadSajjad.Ghaemi@nrc-cnrc.gc.ca (M.S.G.)

In brief

Using machine learning to map immune interdependencies, this study deploys random forests (RFs) to compare vaccine-induced immunity in people with HIV versus HIV-negative age-matched controls across up to five SARS-CoV-2 doses. RFs reveal distinct saliva- and blood-based humoral patterns and a subset of subjects whose responses resembled controls, signaling partial immunologic restoration. Privacy-preserving synthetic patients enable RF training on synthetic data that generalize to real individuals, supporting precision-guided vaccination and follow-up.

Korosec et al., 2026, Patterns *7*, 101474
March 13, 2026 © 2026 The Authors. Published by Elsevier Inc.
https://doi.org/10.1016/j.patter.2025.101474

CellPress

Patterns

CellPress
OPEN ACCESS

Article

Modeling of longitudinal immune profiles reveals distinct immunogenic signatures following five COVID-19 vaccinations among people living with HIV

Chapin S. Korosec,[1,2,3,9,*] Jessica M. Conway,[4] Vitaliy A. Matveev,[5] Mario Ostrowski,[5,6,7] Jane M. Heffernan,[2,3] and Mohammad Sajjad Ghaemi[8,*]
[1]Department of Mathematics and Statistics, University of Guelph, Guelph, ON, Canada
[2]Modelling Infection and Immunity Lab, Mathematics and Statistics, York University, Toronto, ON, Canada
[3]Centre for Disease Modelling, Mathematics and Statistics, York University, Toronto, ON, Canada
[4]Department of Mathematics, Pennsylvania State University, University Park, PA, USA
[5]Department of Medicine, University of Toronto, Toronto, ON, Canada
[6]Department of Immunology, University of Toronto, Toronto, ON, Canada
[7]Keenan Research Centre for Biomedical Science, St. Michael's Hospital, Unity Health, Toronto, ON, Canada
[8]Digital Technologies Research Centre, National Research Council Canada, Toronto, ON, Canada
[9]Lead contact
*Correspondence: chapinskorosec@gmail.com (C.S.K.), MohammadSajjad.Ghaemi@nrc-cnrc.gc.ca (M.S.G.)
https://doi.org/10.1016/j.patter.2025.101474

THE BIGGER PICTURE Vaccines are central to pandemic control, yet immune responses can vary greatly between individuals and populations. People living with HIV (PLWH) represent an important group for whom vaccine efficacy and immune durability remain under-characterized. By utilizing machine learning to profile vaccine-elicited biomarkers (such as cytokines and antibodies), this study identifies how COVID-19 vaccination elicits distinct immune response signatures in PLWH versus HIV-negative participants. Specifically, it shows that cytokines in combination with saliva antibodies best distinguish PLWH vaccine responses, suggesting functional differences in T cell activation as well as mucosal immune response. These results provide a mechanistic framework for understanding variable vaccine outcomes in immunocompromised populations and offer a model adaptable to other diseases and cohorts. More broadly, this work illustrates how data-driven immunology can inform precision vaccination strategies and improve continuity of care in immunologically compromised groups.

SUMMARY

The immune response to vaccination is highly heterogeneous and arises from a dynamic interplay of immune components. Harnessing machine learning (ML) to learn immune interdependencies offers the potential not only to decode immune signatures linked to a specified comorbidity but also to reveal individualized patterns laying the groundwork for precision-guided vaccination and targeted clinical follow-up. We employ a random forest (RF) approach to classify informative differences in immunogenicity between older people living with HIV (PLWH) on antiretroviral therapy (ART) and an age-matched control group who received up to five SARS-CoV-2 vaccinations. RFs identify evidence for T helper 1 (Th1) imprinting and reveal novel distinguishing immune features, such as saliva-based antibody screening, as promising diagnostic tools (whereas serum IgG is not). Our modeling approach reveals a subset of PLWH whose immune signatures are indistinguishable from the HIV− control group, which we interpret as near-complete immune restoration from a longitudinal vaccine-elicited immunogenic perspective. To expand the utility of our findings, we generate privacy-preserving synthetic "virtual patients" that accurately approximate the original longitudinal immunologic data and show, via train-on-synthetic/test-on-real evaluation, that RF classifiers trained solely on virtual patients generalize to held-out real patients. Our results highlight the effectiveness in utilizing informative immune feature interdependencies for classification tasks and suggest broad impacts of ML applications for personalized vaccination strategies among high-risk populations.

© 2026 The Authors. Published by Elsevier Inc.
This is an open access article under the CC BY license (http://creativecommons.org/licenses/by/4.0/).

INTRODUCTION

HIV remains a global health burden, with approximately 40 million current infections (people living with HIV, PLWH) and more than 40 million deaths to date.[1] Given the current global trends in new infections, millions of new infections are expected by 2050.[2] Antiretroviral therapy (ART) has significantly improved life expectancy and overall health outcomes for PLWH.[3,4] However, effective HIV viral load suppression may not lead to full recovery[5–8]; further, individuals with advanced HIV disease are susceptible to more severe COVID-19 outcomes.[9] Vaccines are the premier prophylactic public health intervention to reduce infectious disease severity and morbidity.[10] The immunogenic response to SARS-CoV-2 vaccines among PLWH depends on vaccine type, disease progression, and ART adherence.[7,11] Machine learning (ML) holds significant promise for revolutionizing HIV diagnostics by uncovering complex immune patterns and facilitating precision health interventions and mobile health diagnostics.[12–14] An ML framework capable of learning the complex humoral and cellular immunological signatures from repeated SARS-CoV-2 vaccine responses in ART-treated PLWH offers a powerful data-driven approach to understand how vaccine immune responses vary across the spectrum of HIV disease progression and would be a significant advancement toward precision-guided vaccination strategies and targeted clinical monitoring.

Longitudinal vaccine-induced immunogenicity against vaccine-preventable infections has been studied in the context of immunodeficiencies in ART-suppressed PLWH for different pathogens and vaccine types.[15,16] Vaccine-elicited responses in ART-suppressed PLWH have often been found to be inferior to those of HIV-negative control groups, although the gap is often diminished following a multidose vaccination regimen.[17] Studies of variations in the humoral and cellular immune responses from repeated vaccination in PLWH and non-infected age-matched controls may shed light on the effects of persistent immune activation due to ART-suppressed HIV infection on the adaptive immune response dynamics. This knowledge could inform customized vaccination strategies,[18] aid in the development of adjuvant therapies,[19] and reduce the risk of severe outcomes[9] in the PLWH immunologically vulnerable group.

Induction of the adaptive immune response to vaccines and pathogens requires the activation and proliferation of $CD4^+$ T helper cells that subsequently activate and generate cellular and humoral immunity.[20,21] The cellular and humoral components of the human immune system can be highly variable across a population, and they can exhibit complex time-sensitive interdependent immune responses to antigenic perturbations.[22] Among PLWH on ART, ART adherence and immune status (CD4 immune responder [IR] versus immune non-responder [INR]) may have an influence on the durability of humoral and cellular responses to SARS-CoV-2 vaccinations.[23] For example, humoral outcomes among PLWH with undetectable plasma HIV viral loads and high CD4 counts have been found to be similar to those of HIV-negative controls for the ChAdOx1 nCoV-19 vaccine,[24] heterogeneous multidose vaccine regimens,[7,25,26] and mRNA-based vaccines[27,28]; however, PLWH classified as INR display CD8 and CD4 responses that significantly differ from those of controls,[7,23] with HIV-related T cell dysfunction associated with less humoral immunity and faster waning.[29] Given enough data, ML algorithms can capture and learn the immune signatures of PLWH versus a control group and, furthermore, may identify which variables are particularly informative or non-informative in distinguishing each individual's immune class. An advantage of ML approaches is that they can reveal complex non-linear relationships between cellular and humoral immune responses without requiring explicit assumptions about the nature of the immunological feature relationships.[30]

The motivation for employing ML approaches is to create an advanced tool capable of processing complex datasets at the individual level. We aim to leverage ML to classify individuals with a defined probability while offering clear, data-driven explanations for each classification decision based on their personal data. With a successfully trained ML tool, consistently *misclassified* PLWH may represent individuals with atypical immune responses to HIV. These responses could include unusually effective viral suppression, the presence of unique genetic factors such as in elite controllers, or intriguing immunological variations in treatment responses. Conversely, age-matched HIV-negative individuals consistently *misclassified* as HIV positive exhibit vaccine-elicited immune profiles resembling those of HIV-positive individuals. Such profiles could arise due to other previously unknown comorbidities, including chronic inflammation, autoimmune diseases, or concurrent infections. ML offers a powerful framework for personalized vaccination strategies in immunology by enabling the classification of individuals based on complex immune signatures while providing interpretable insights into atypical responses. By identifying misclassified cases, we can uncover novel immunological variations, including unique host factors in PLWH and previously unrecognized comorbidities in HIV-negative individuals, ultimately refining precision medicine approaches for vaccine response assessment.

In the following, we investigate heterogeneity in the immune response from SARS-CoV-2 vaccine-elicited immunological outcomes among ART-suppressed PLWH who received up to five doses of the SARS-CoV-2 vaccines over 104 weeks. The HIV-specific responses are classified against an age-matched control group, thereby controlling for known age-related immunological variations and dysregulations.[31] The dataset that we study is an extended dataset from Matveev et al.[7] We find that random forest (RF), which is capable of learning complex non-linear interdependencies between immunological features that traditional statistical and mechanistic models may have missed or are incapable of learning,[32] is able to distinguish between the PLWH and the age-matched control immune responses with near-perfect accuracy, given particular biomarkers. Our findings reveal that there are informative and non-informative immunogenic features implicated in the adaptive immune response of PLWH; informative features are determined by the RF to be very important in classifying immune responses, while non-informative immune features provide little information toward classifying the immune responses. For instance, while saliva and serum IgGs were non-informative, saliva IgA emerged as a critical indicator in identifying HIV-modulated immunogenic responses; further, cytokine and saliva IgA features in combination are found to form an optimally stratified feature subset leading to performance metrics equivalent to those of the full dataset.

CellPress
OPEN ACCESS

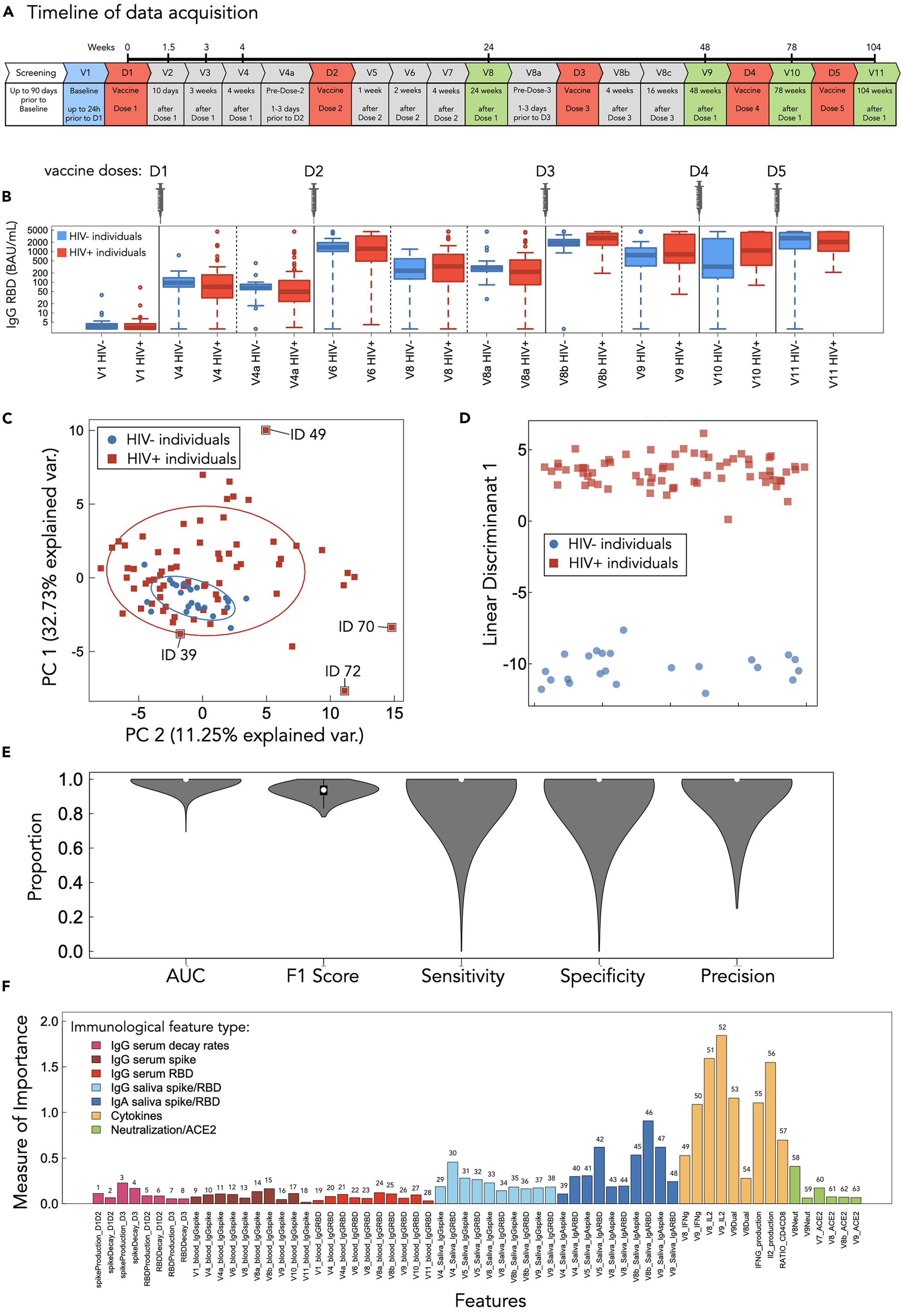

Figure 1. Dataset overview and RF performance metrics

(A) Timeline of study and data acquisition.

(B) IgG RBD features for the HIV− and HIV+ classes for doses 1 through 5 visualized as boxplots.

(legend continued on next page)

Finally, we extend our study to generate synthetic datasets that reproduce the local and global characteristics of the human-based dataset. We employ both supervised and unsupervised synthetic data generation methods, such as multivariate normal (MVN), Gaussian mixture model (GMM), synthetic minority oversampling technique (SMOTE), and K-nearest neighbors (KNN). We demonstrate that the preservation of local or global data characteristics depends on the synthetic data generation method. Finally, we assess how well each synthetic dataset performs with RF classification and discuss future considerations.

RESULTS

To characterize the immunogenic responses elicited by repeated SARS-CoV-2 vaccinations in ART-treated individuals with HIV (PLWH) versus an age-matched control group, we employed a combination of unsupervised and supervised ML methods. Principal-component analysis (PCA) and linear discriminant analysis (LDA) were utilized to explore data clustering and class separability, respectively. An RF classifier was then implemented to identify complex, non-linear immunogenic signatures that differentiate the groups, while feature importance analyses indicated key immunological contributors to these differences. t-stochastic neighbor embedding (t-SNE)-based correlation network visualization was then employed to enhance our understanding of the feature interrelationships. These approaches collectively provided a comprehensive framework for uncovering meaningful immunological patterns and for classifying responses with high accuracy. Finally, we utilized various supervised and unsupervised ML methods to accurately capture the local and global data characteristics and demonstrate that synthetic data can reproduce the clustering and RF classification behavior as the original dataset.

Study data timeline and participants

Our data are an extended dataset from our previous study[7] to include doses 4 and 5 of COVID-19 vaccination. In total, 91 participants were recruited into the study—23 HIV-negative individuals and 68 PLWH on ART. The timeline of the clinical study is shown in Figure 1A. Study participants were given five vaccine doses, and biomarker measurements were made in each study interval, see methods and Matveev et al.[7] for details. The full dataset used in this work comprises 63 immune features, consisting of serum and saliva IgG, saliva IgA, IFN-γ- and interleukin-2 (IL-2)-producing T cells, and dual-responding IL-2/IFN-γ cells, CD4/CD8 ratio, virus neutralization, and ACE2 displacement, drawn from individuals throughout the course of their multivaccine regimen across the study timeline (Figure 1A). Figure 1B plots the raw IgG RBD data.

SARS-CoV-2 vaccine responses of PLWH and controls can be classified with near-perfect performance

PCA is an unsupervised method that can be used to reduce the dimensionality of high-dimensional data by identifying the directions (principal components) that capture the most variance in the dataset. PCA can thus be used to learn whether vaccine-elicited immune responses among PLWH versus the age-matched controls differentiate the classes effectively. The PCA results do not show clear separation between the groups (Figure 1C). However, we observe that HIV− individuals subcluster within the HIV+ cluster (Figure 1C). Additionally, we find that PLWH exhibit a broader spread along both principal components 1 and 2 (PC1 and PC2, respectively), evidenced by the 95% confidence ellipses. We include labels for outlier study participant IDs 49, 70, and 72; we interpret outliers as individuals who may have distinctive immune responses.

In order to maximize class separation and check for separability, LDA, a supervised and linear dimensionality reduction technique that focuses on maximizing the separation between predefined classes, was employed. The LDA results using the first linear discriminant (LD1) are shown in Figure 1D and present a clear separation between the HIV− and the HIV+ groups. We thus conclude that the immunological features included in our study can be used to discriminate between the HIV- and the HIV+ classes.

While PCA and LDA provide an intuitive visualization of data to explore class separability, these methods are not able to capture the complex interdependencies between features. However, the class separability pointed to by LDA suggests that a more advanced algorithm may be able to learn unique immunological signatures that capture the PLWH immune response that distinguishes it from the control population. We therefore used an RF classifier to characterize the complex non-linear signatures differentiating HIV+ and HIV− immunological responses in order to enhance predictive accuracy. This can also provide mechanistic insights on the underlying immunological trends. The details of the RF implementation can be found in the methods. We benchmarked RF against simpler linear and kernel methods under the same stratified 5-fold outer CV with inner-fold tuning where applicable: logistic regression, LDA, linear SVM, RBF SVM, and greedy forward/backward logistic feature selection (area under the curve [AUC]-driven). As summarized in Table S1, RF achieved the strongest, or near strongest, overall metrics (AUC-receiver operator curve [ROC], AUC-PR [precision-recal], F_1, and accuracy) relative to these baselines.

The RF models achieved a perfect median AUC-ROC (area under the receiver operator curve; measures a model's ability to distinguish between classes, evaluating performance across all classification thresholds) of 1.0 (Figure 1E). AUC-ROC reflects the model's ability to distinguish between the HIV-positive and the HIV-negative classes across all possible classification thresholds. The AUC-ROC median value of 1.0 suggests that the RF classifier has excellent overall classification performance on our immunological dataset.

The RF model's median F1 score (the harmonic mean of precision and recall, balancing false positives and false negatives) was found to be 0.94 (Figure 1E). This reveals a trade-off between precision (the proportion of correctly identified positive

(C) PCA with the first two largest components shows that significant source of variation in the data is not driven by the HIV+ group.
(D) The first largest component (LD1) of LDA reveals the existence of HIV-specific signatures that are not distinguishable through the unsupervised PCA.
(E) The performance metrics—AUC-ROC, F1 score, sensitivity, specificity, and precision—demonstrate the strong predictive power of the RF model.
(F) The measure of importance, as assessed by the RF algorithm, is shown for all 63 features. Features are grouped by immunological type and color coded.

instances) and recall (the proportion of actual positives correctly identified) and suggests that there may be a moderate number of false positives (affecting precision) or false negatives (affecting recall) in the predictions. We report median values of 1.0 for sensitivity, precision, and specificity (Figure 1E), which all suggest that the model often achieves near-perfect performance, but considerable variance around these median values implies that this performance is not consistent across all data folds.

AUC-ROC and F1 score performance measures from RF trained on individuals with randomized labels are all found to be ∼0.5 on median (Figure S1). To further explore RF classification heterogeneity for each individual, as well as the likelihood of HIV+ classification across all models, we provide all probability distributions among all IDs in Figures S2A and S2B.

We conclude that our implementation of RF is generally effective at classifying vaccine-elicited immunogenic responses among PLWH versus the age-matched control group. Further exploration of the RF results and the significance of the outliers is discussed below.

RF reveals T cell responses to be most important features in distinguishing the vaccine-elicited immune response between PLWH and the age-matched HIV– control group

Successfully mounting, and maintaining, vaccine-elicited immunity involves a myriad of immunological components (for a good review see Pollard and Bijker[20]). Immunodeficient states, such as those that can be found in ART-suppressed PLWH, can have complex dysregulated immune signatures—where particular components of the immune response can be dysregulated more than others. In our feature space, we are therefore interested in using the RF algorithm to suggest which immune components are informative in distinguishing the PLWH SARS-CoV-2 vaccine-elicited responses from the age-matched controls. Such insights may be useful toward forming mechanistic hypotheses for how ART-suppressed HIV infection affects adaptive immune system dynamics.

To assess how each feature contributes to RF model accuracy we computed the feature importance (see methods for details). We plot the importance estimates in Figure 1F for all 63 immune features. Features with higher importance scores have a more significant impact on improving model predictions in decision tree splits that underlie the RF, suggesting they capture significant information that can differentiate the classes. Our analysis indicates that the frequencies of SARS-CoV-2 spike-specific T cells measured by cytokine production are generally the most important features, with the three IL-2 features (visits 8 and 9 and IL-2 production rate) having the highest importance over all other features. Conversely, the serum-based humoral features all rank as least important, suggesting information contained within these immune features is redundant toward classification. Below, the importance rankings (Figure 1F) will be used in forward and reverse ablation analyses to determine if there exists a minimally stratified combination of features that leads to optimal RF performance.

t-SNE reveals that important features tend to form clusters

We use t-SNE to visualize the structure of our feature dataset. Figure 2A presents the correlation network of the dataset using all 63 biomarkers, where circle size represents the corresponding biomarker RF importance. Here, we learn that IgA, cytokines, and IgG biomarkers tend to cluster together, but the cluster densities may increase if there are more important biomarkers within that class. We also learn that biomarkers that are determined to be important (e.g., IL-2 and IFNs) tend to form distinct groupings, suggesting correlated expression patterns, while features that are determined to be unimportant (e.g., spike and RBD IgG) disperse, suggesting weaker correlations among these features. Figures 2B and 2C display the same t-SNE layout as Figure 2A; however, only significant edges connecting to a cytokine feature are displayed, where color is now used to illustrate the clear and opposing RF feature weight trends found for HIV− (Figure 2B) and HIV+ (Figure 2C) individuals. From this we can see that there are clearly opposing RF weights assigned to the cytokines, depending on whether the individuals are PLWH or age-matched controls.

The t-SNE layout reveals that the most important features identified by the RF algorithm form distinct clusters, suggesting that these features not only drive the predictive power of the RF model but also represent well-defined biologically meaningful patterns in the data (Figure 2A), with the RF oppositely weighting key features to distinguish the classes (Figures 2B and 2C). We next plot the full distributions for all feature weights across all folds to visualize the distributions of RF weights. The feature weight distributions from all folds from all ($n = 23$) control individuals and from all ($n = 68$) PLWH are shown in Figures 2D and 2E, respectively. We see more clearly that classification of the control class is highly dependent on the cytokine measures, which have negative weights on median. There is very little weight placed on any of the other features except V8B IgA RBD saliva, whose weight distributions deviate below 0 on median. Conversely, for PLWH, we find that cytokine features are positively weighted (Figure 2E, yellow features). We also find the V8B IgA RBD saliva values to be positively weighted, opposite those of the control. While the median weights for all other features are ∼0, we observe significantly more dispersion in median values for the PLWH compared to the control (Figure 2E, all non-yellow features). As validation to confirm the stability and biological relevance of the t-SNE clusters, we perform uniform manifold approximation and projection (UMAP) on our data using as the nearest-neighbor parameter the t-SNE perplexity value. The results are shown in Figure S12, where similar feature clustering behavior compared with t-SNE is found.

Figure 2D shows that the control class is relatively homogeneous (most feature weights are close to 0) and that only cytokines are needed to distinguish HIV− individuals from the HIV+ class. Figure 2E shows significant RF feature weight variance for the HIV+ class for serum and saliva IgG biomarkers in addition to heavily weighted cytokine features, suggesting that many more features are needed, potentially in combination, for correct classification. The significant dispersion found in Figure 2E suggests heterogeneity in disease pathology and immune responses across PLWH, indicating that, due to the high variability and complexity of the HIV+ immune response, a diverse set of trees in the forest is required to accurately classify instances of the HIV+ class, where each tree may rely on different combinations of features. Therefore, we next study the RF performance under various ablation procedures to determine a

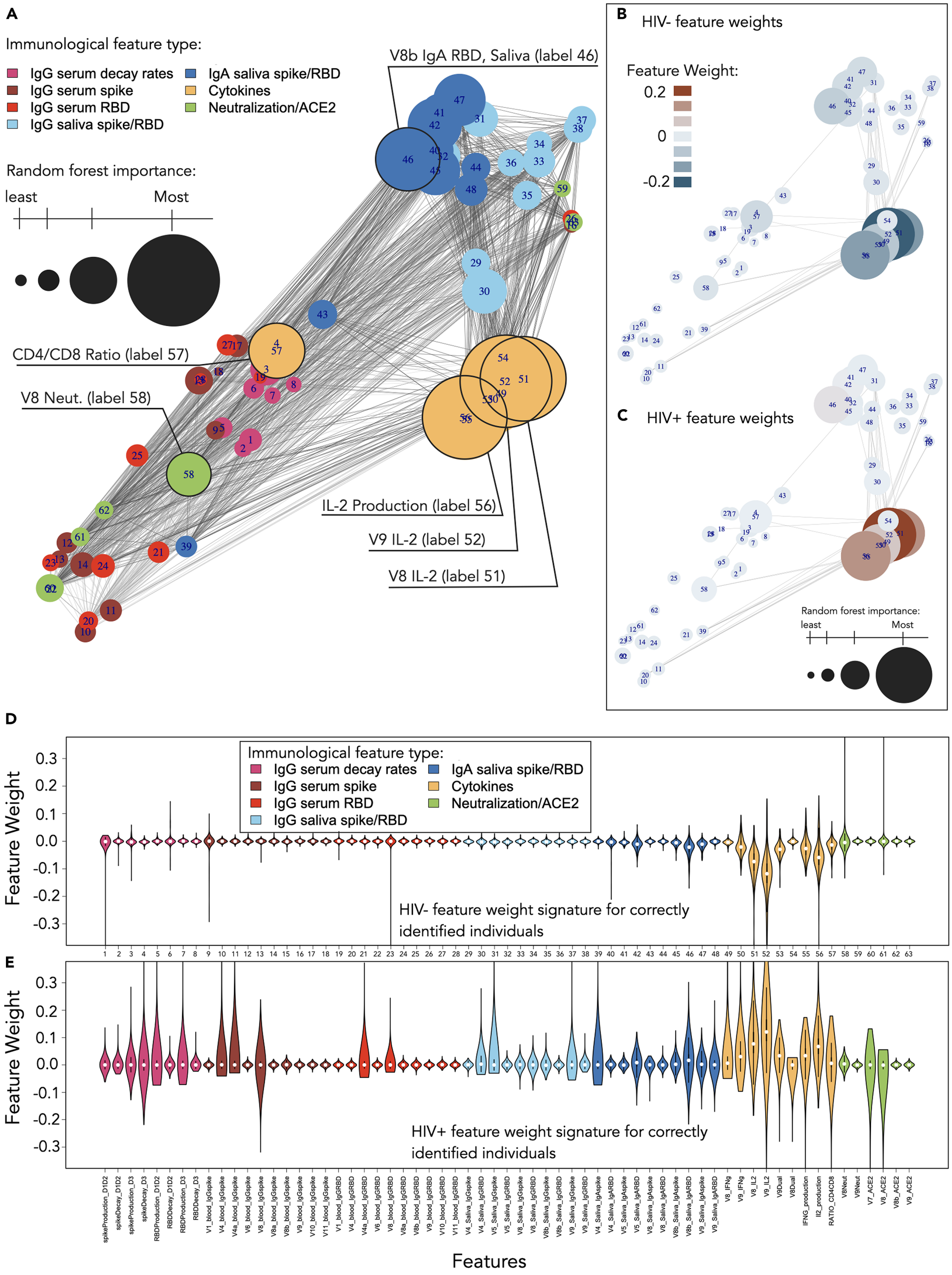

Figure 2. Correlation network and feature weight distributions

(A) A correlation network of the 63 immunological features is visualized using t-SNE applied to the adjacency matrix to calculate the layout. Features are colored by type, and node sizes scale relative to the importance measures from Figure 1F. For visualization, each feature is represented as a node. An edge is shown between a pair of nodes when their correlation is statistically significant (*p* values < 0.05).

(B and C) The subsets of network connections with statistically significant correlations, which further depend on the cytokine features, are shown. Here, the color scale corresponds to the mean feature weights for HIV− (B) and HIV+ (C) individuals.

(D and E) The distributions of RF feature weights computed across all models are shown for the individuals who are correctly classified more than 95% of the time. Median cytokine feature weights (yellow) clearly form opposite trends when comparing HIV− (D) and HIV+ (E) RF results.

CellPress
OPEN ACCESS

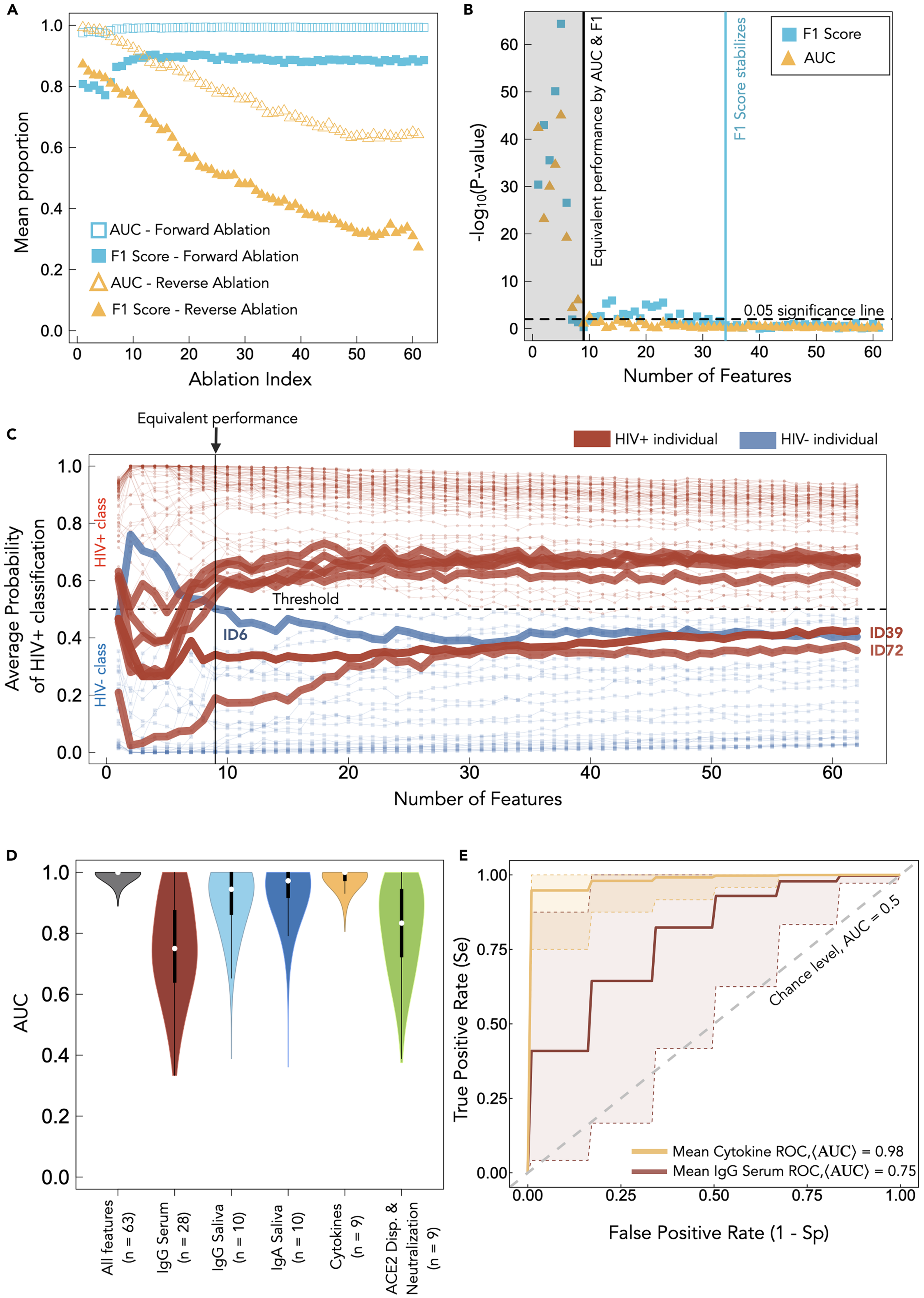

Figure 3. Ablation analyses and model reduction

(A) Mean AUC-ROC and F1 score are shown as a function of the ablation index for the forward and reverse ablation analyses.

(B) *p* values from the Wilcoxon signed-rank test performed between the full-feature RF model and each of the performance measure distributions from the forward ablation procedure are shown and BF corrected.

(legend continued on next page)

minimum stratified set of features for optimal classification. We also explore sensitivity to RF performance with various combinations of features by their clinical type.

Ablation analysis reveals an optimally stratified feature subset for classification

Ablation analysis identifies which features are essential for classification and which are redundant or irrelevant. Identifying a minimally stratified dataset reveals the key immunological markers (e.g., specific cytokines or antibodies) that distinguish HIV+ from HIV− individuals and, thus, can enhance understanding of the biological mechanisms driving the immune response in HIV infection. Further, the immunological dataset may include noisy features. Identifying and removing these features can improve model clarity and reduce overfitting.

To identify the minimal set, we perform both forward and reverse ablation (see methods). The results are displayed in Figure 3A. Forward ablation (blue hollow squares) reveals that just the top two features (V8 and V9 IL-2 measures) can be used to produce a median AUC-ROC just below 1.0. Conversely, reverse ablation results in a monotonic decrease in median AUC-ROC (yellow hollow triangles) down to a minimum median of ∼0.65. The final two features identified via reverse ablation are the visit 11 blood IgG spike and visit 11 blood IgG RBD features.

Note that the forward ablation F1 score follows a non-monotonic relationship as a function of forward ablation index (blue filled squares). The top two features produce an F1 score of ∼0.8 (forward ablation index 1, first blue square in Figure 3A), but the F1 score decreases to a minimum of ∼0.76 by forward ablation index 6 before increasing up to a plateau of ∼0.9 as additional features are added. As a function of reverse-ablation index, the F1 scores (yellow filled triangles) decrease, as expected, to a minimum of ∼0.3. Thus, the two least important features lead to F1 score of 0.3 and AUC-ROC of 0.7 (Figure 3A solid and hollow yellow triangles, respectively).

Figure 3B demonstrates the quality of the identified minimal set. A Wilcoxon test is used to compare the AUC-ROC and F1 score distributions from the forward-ablation algorithm to the full 63 feature distributions (shown in Figure 1E). Considering a *p* value of 0.05 to be the significance threshold, we find the top 9 features' performance to be statistically similar to the complete 63 feature dataset, with both the AUC-ROC and the F1 scores used for comparison. Thus, these 9 features may constitute an optimally stratified feature set for classifying SARS-CoV-2 vaccine immune responses between HIV+ and HIV− immune statuses. Note that, following the ninth forward ablation index, the AUC-ROC remains under the 0.05 significance line (Figure 3B, yellow triangles), while the F1 -score-associated *p* value increases to above significance and does not settle to below significance until the 34th feature is added (Figure 3B, blue squares). Therefore, the addition of more features beyond the optimally stratified set of features adds redundant information and can lead to a tendency to misclassify some individuals. In decreasing order of importance, the 9 features in the optimal set are visit 9 IL-2, visit 8 IL-2, the IL-2 production rate, visit 9 dual responding cells, the IFN-γ production rate, visit 9 IFN-γ, visit 8b saliva IgA RBD, the CD4/CD8 ratio, and visit 5 saliva IgA RBD. For clarity, the IL-2 and IFN-γ production rates refer to the rate of change of the increasing frequencies of cytokine-producing T cells, as a measure of spike-specific T cells, during the course of the booster series, calculated in our previous work.[7] Thus, 7 of the top 9 features are cytokine based, while 2 are saliva IgA RBD-based features.

Saliva humoral responses are good predictors of PLWH SARS-CoV-2 vaccine immunogenicity

Figure 3D shows the AUC-ROC distributions of RF performance for RF models trained according to the characteristics of their clinical type, i.e., serum IgG, saliva IgG, saliva IgA, cytokines, and neutralization/ACE2 displacement. Figure 3E displays the mean ROC curves with 95% confidence intervals for cytokines (best performing feature family) and serum IgG (worst performing feature family). In Figure S5, we provide the accompanying mean PR plot for the cytokine and serum data. These results show that the RF model trained on cytokine features provides a near-perfect classifier. The IgA and IgG saliva features then take second and third place, in terms of AUC-ROC performance, with median AUC-ROC values of 0.98 and 0.95, respectively. Classification performance then drops when using ACE2 displacement/neutralization and IgG serum features to median values of 0.83 and 0.75, respectively. The IgG serum features thus provide a near-baseline classifier. There is large dispersion (standard deviation) in AUCs found for the last two clinical feature classes of 0.14 and 0.16, respectively. The significant biological ramifications of this result, specifically that mucosal IgG and IgA appear to be highly informative, while serum IgG is not, are highlighted in the discussion in the context of known HIV-related mucosal immune dysregulatory mechanisms.

Misclassified individuals suggest atypical vaccine immune responses

The identification of individuals whose immunological responses lead to classification errors may suggest closer follow-up by clinicians. For example, consistently misclassified PLWH may represent atypical immune responses to HIV, such as unusually effective viral suppression, unique genetic factors (e.g., elite controllers), or interesting immunological variations in treatment responses. Conversely, age-matched HIV− individuals consistently misclassified as HIV+ might have immune profiles resembling those of HIV-positive individuals due to other comorbidities, such as chronic inflammation, autoimmune diseases, or infections. To further investigate the non-monotonic F1 scores, we identify which individuals are contributing to the initial decline in F1 score shown in Figure 3A. Figure 3C provides the average

(C) Average probability trajectories for all 91 individuals as a function of number of features from the forward ablation algorithm. Multiple individuals whose probability of classification crosses over the threshold at index 9 are shown at the equivalent performance mark (solid line).
(D) AUC-ROC distributions of RF performance are shown for RF models trained on features in isolation by their clinical type. Here, *n* refers to the number of features of each type.
(E) Mean ROC curves generated using predictions from the test set for RF models trained on cytokine (yellow) and IgG serum (red) data types are shown. Shaded regions corresponding to 95% confidence intervals are highlighted by a dashed line.

probability of HIV+ classification for all individuals as a function of forward ablation index. We highlight a number of trajectories that, by index 9 (the equivalent performance index), cross the classification threshold. The visualization provided in Figure 3C provides a deeper intuition of the model performance metrics displayed in Figures 3A and 3B. IDs 39 and 72, two PLWH, are the only IDs to remain in the incorrect region beyond forward ablation index 9 and remain so when all features are present. IDs 39 and 72 are misclassified in 95% and 85% of the RF iterations, respectively, whereas seven individuals are misclassified in 10%–50% of the RF iterations. The remaining 82 individuals are classified correctly in nearly 100% of the RF iterations. Figure S2 provides an additional visualization displaying the probabilities of HIV classification for all individuals in the study as well as ID 39's unique immunological weight signature, which appears similar to the HIV− control signature.

Figure S2B displays the HIV+ classification probabilities for all 91 individuals. In Figure S2C, we include the individual feature weight distributions, across all RF models, for ID 39, who is an HIV+ individual misclassified 95% of the time. ID 39's cytokine feature weight signature matches the HIV− feature signature shown in Figure 2D. Further, an example of a single training landscape from a randomly selected RF model is shown in Figure S3, with two arbitrary study participant ID examples illustrated in Figure S4. Altogether, these results suggest there may be redundant features present that may mislead the RF algorithm, or there are outlying individuals with unique signatures (e.g., ID 39) that result in the RF algorithm placing significant weight on non-cytokine features. Using the available clinical metadata on years of viral-load suppression (Table S3), neither ID 39 nor ID 72 appeared atypical: both durations (12 and 14 years, respectively) were slightly below the cohort median/mean (18.4/19 years), and neither was flagged by a non-parametric percentile test (empirical two-sided $p > 0.05$) or by a robust Z score (MAD scaled; $|Z| < 3$); their values were also well above the cohort minimum ($\sim$5 years).

Synthetic data provide a physiologically accurate representation of immunological data

We implemented Gaussian mixture models (GMM), multivarient normal (MVN) distribution, synthetic minority over-sampling technique (SMOTE), and K-nearest neighbors (KNN) methods in order to generate synthetic data. We ensured that the generated data matched the original dataset in size for all iterations: 64 features total, comprising 23 HIV− individuals and 68 PLWH. Detailed descriptions of all ML algorithms performed can be found in the methods.

GMM leads to the lowest mean Kullback-Leibler divergence (KLD) and second-lowest KLD variance, followed by the MVN approach (Figure S6). By KLD metrics, the worst performance is found to be SMOTE, closely followed by KNN (for all three k values examined). Figure 4A shows each calculated KLD value for all 63 features for the GMM approach, where a broad distribution of KLD values as a function of feature type is revealed. Figure 4A prompted further analysis to determine whether some synthetic generating techniques are better for specific features than others. In Figure S7, we provide a t-SNE plot with the same layout as in Figure 2A, where color corresponds to the synthetic data generating technique that produced the lowest KLD for each feature. We find no discernable relationship between synthetic generation method and feature cluster behavior.

Figure S6C displays the result of projecting synthetic-feature principal components onto the feature principal components of the original data. Here, a synthetic technique that leads to distinct clustering from the original PCA suggests that the features do not preserve the local structure. We find that all synthetic datasets produce nearly overlapping ellipses by this PCA. Individual PCAs performed independently on each synthetic matrix are shown in Figure S8. Comparing the PCAs in Figure 1C, we find that the supervised synthetic approaches SMOTE and KNN (with k = 20) reproduce the PCA subclustering pattern. This presents an interesting juxtaposition between two different methods of evaluating synthetic data: one based on a quantitative measure (KLD) and another based on a visual/qualitative assessment (PCA plot comparison). The KLD analysis suggests that GMM is capturing the overall statistical properties of the data the best (SMOTE being the worst), while the PCA suggests that data generated by SMOTE and KNN (with k = 20) better capture the cluster structure when the data are projected onto the first two principal components.

Figure 4B shows the importance measures of all synthetic datasets used in the RF models, with the RF model training performed similarly to that in the previous sections (see methods). We find that GMM and MVN lead to overemphasized importance for visit 4 saliva RBD IgG, visit 4 serum IgG RBD, and a visit 1 serum IgG RBD. SMOTE appears to perform overall quite well in terms of reproducing the importance feature distribution, with all RF feature importance metrics comparing best to the original data.

Synthetic data generalize well to real-patient data: TSTR

To directly test whether models trained entirely on synthetic data generalize to real patients, we used a train-on-synthetic, test-on-real (TSTR) protocol. Synthetic training sets were generated per fold with a class-conditional GMM (cGMM) fit on the training fold only (leakage free) and evaluated with RF on the held-out real fold with real-trained RF serving as baseline. Aggregated over all folds, the synthetic-only RF achieved AUC-ROC = 0.991 and AUC-PR = 0.997, with accuracy = 0.928 and F1 = 0.955 on real patients, just less than the real-trained baseline (AUC-ROC = 0.996, AUC-PR = 0.999, accuracy = 0.945, F1 = 0.97). Feature-importance profiles were mechanistically concordant, with a mean Spearman $\rho = 0.736$ between permutation-importance vectors from synthetic-trained and real-trained RFs (Figures 5A and 5B), with Figure 5C showing the per-fold $\times$ repeat differences (synthetic-only minus baseline) for each performance metric, demonstrating overall good agreement between synthetic and actual RF-trained models. Both models were evaluated on the same held-out real fold. Metrics were AUC-ROC (pROC), AUC-PR (PRROC), accuracy, and F_1. Figure 5C shows the distributions of the per-fold $\times$ repeat differences, Δ = (synthetic only) $-$ (baseline), for each metric.

DISCUSSION

Our implementation of RF is capable of accurately classifying the SARS-CoV-2 vaccination response of PLWH from an age-matched HIV-negative group with humoral and cellular immunological data from SARS-CoV-2 vaccination. Our ablation

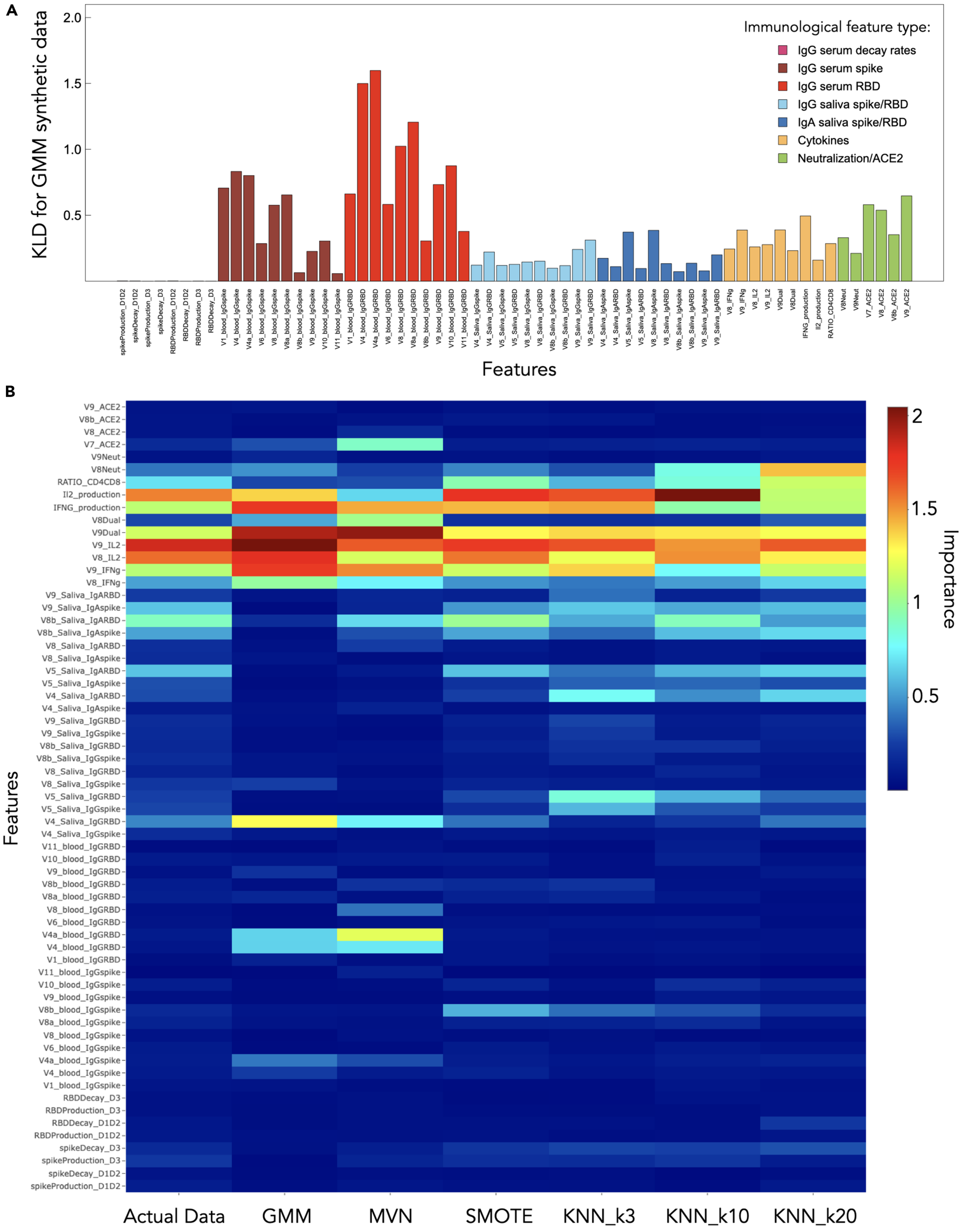

Figure 4. Synthetic data analysis

(A) KLD is shown for each feature for the GMM synthetic data, which provided the lowest mean KLD and second-lowest total KLD variance.

(B) Heatmap of the RF importance for all features of the original data (reproduced from Figure 1F) and all synthetic data-generating approaches.

procedures, based on feature importance, reveal an optimized subset of features, comprising IL-2 and IFN-γ cytokine-producing T cells, the CD8/CD4 ratio, and saliva IgA RBD, that lead to RF model performance equivalent to that of the full 63 feature dataset (Figures 3A–3C). Thus, cytokine-based features in combination with post-booster saliva IgA features contain most of the predictive power to differentiate PLWH immunogenic responses in our dataset. Adding additional features beyond the optimal 9 appears to have little effect on the AUC but causes the F1 score distribution to deviate from the full-feature model

CellPress
OPEN ACCESS

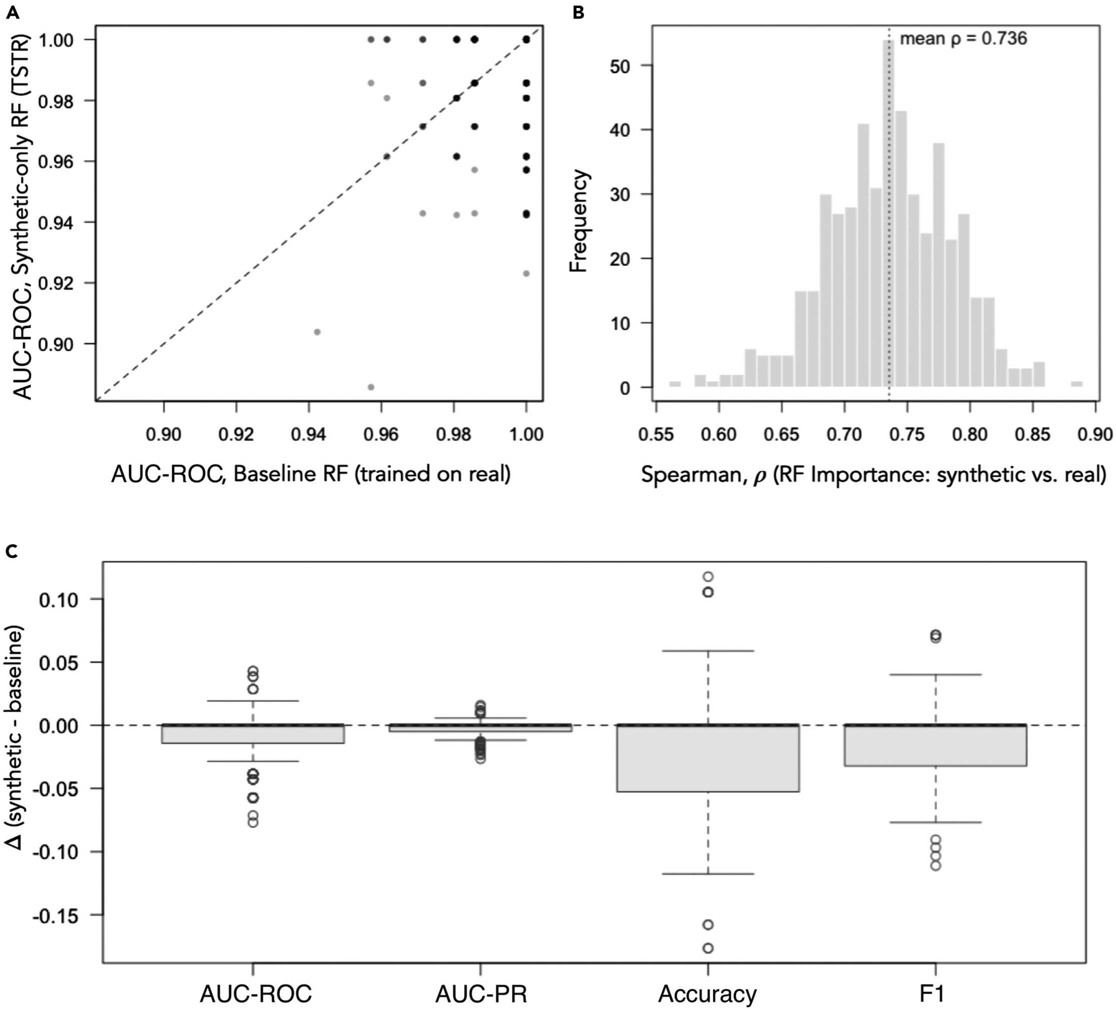

Figure 5. RF model trained entirely on synthetic data generalizes to real patient data

(A) Parity of AUC-ROC between a real-trained baseline RF and a model trained exclusively on conditional GMM-generated synthetic data (TSTR); points are fold evaluations and the dashed line is $y = x$.

(B) Distribution of Spearman rank correlations between permutation-based feature-importance vectors from synthetic-trained and real-trained models (dashed line is the mean).

(C) Performance deltas for TSTR with class-conditional GMM synthesis. Boxplots show per fold-repeat differences on the held-out real folds for four metrics: AUC-ROC, AUC-PR, accuracy, and F1. The dashed horizontal line marks zero (parity). Boxes denote the median and interquartile range; whiskers extend to $1.5 \times$ IQR; points are outliers.

F1 score distribution until the 35th feature is added. We believe this to be a commonly observed phenomenon in which additional features beyond the optimal subset confuse the model and degrade performance.[33] Thus, the ablation procedure finds that the 9 optimal features contain most of the classification power, with the remaining 54 features contributing mostly minimal or redundant information. The eventual improvement at 35 features (Figure 3B, blue squares) indicates that additional useful information is present but becomes beneficial only when enough features are considered together, suggesting that some further interactions or combinations of immunological responses are relevant.

Analyzing individual classification probabilities reveals there is a select group of individuals that the RF algorithm has difficulty correctly classifying (IDs 39, 72, and 6 from Figure 3C). Consistently misclassified PLWH may reflect atypical immune responses, such as elite controllers or unique treatment effects, while misclassified HIV-negative individuals may have immune profiles resembling HIV-positive cases due to comorbidities like chronic inflammation or autoimmune conditions; therefore, the RF algorithm can be used to identify these misclassified individuals for closer clinical follow-up to uncover unique immune mechanisms, guide personalized care, and address potential comorbidities or atypical disease progression. This optimization framework lays the foundation for immune system monitoring in longitudinal vaccine studies when balancing comprehensive immunological profiling in resource-limited settings. The resulting sparse model not only preserves the predictive power of

the RF classifier but also delivers a feasible approach for vaccine response when the high costs of clinical studies are challenging. As such, identifying key predictive parameters fosters robust immunological surveillance by simultaneously shrinking model complexity and sampling burden.

We observed that immunological feature types that form distinct clusters by t-SNE, such as cytokines, saliva IgG, and saliva IgA (Figure 2A), also appear to produce relatively high AUC-ROCs when training RF models in isolation (Figure 3D). In contrast, clinical features that do not form distinct clusters by t-SNE, such as serum IgG and neutralization/ACE2 displacement, lead to relatively poor median AUC-ROC performance (Figure 3D). Therefore, there may be an underlying relationship between the highly dimensional t-SNE feature clustering and RF predictive power. t-SNE is an unsupervised method used to visually capture the underlying local and global structure inherent to a dataset.[34] RF models tend to perform optimally when provided with informative non-redundant features.[35] t-SNE, by revealing local cluster formation, may be implicitly revealing subsets of features that have low internal noise and high predictive relevance. Indeed, previous work has successfully implemented an initial t-SNE screening step to eliminate redundant data from a large reference dataset prior to RF model training.[36] Supervised and unsupervised clustering may therefore warrant further exploration in an immunological context to reduce redundancy in datasets prior to model training.

T cell responses play a crucial role in shaping immunity after mRNA COVID-19 vaccination,[37] with spike-specific IFN-γ and IL-2 T cell responses emerging as key factors following repeated vaccination.[38,39] In this work, we use ELISpot cytokine responses to SARS-CoV-2 spike peptides as a measure of frequency of spike-specific T cells. We found the most important features to distinguish PLWH vaccine-specific immunological outcomes in our RF approach to be the post-primary series and booster IL-2 responses and post-booster IFN-γ responses, resulting in a median AUC-ROC of $\sim$0.97 and F1 score of $\sim$0.8 (Figure 3A). Cytokine-based statistical differences between PLWH and the HIV-negative individuals were previously documented with this dataset and found to deviate significantly from the HIV-negative individuals, with the underlying mechanism hypothesized to be T helper 1 (Th1) imprinting from pre-existing HIV infection.[7] Clinicians are more often utilizing ROC curves to make diagnostic judgements.[40] Our ROC (Figure 3E) and PR (Figure S5) curves computed from RF training on cytokine features indeed show the cytokine features to be diagnostically near-perfect classifiers in the context of vaccine-elicited immunity among PLWH.

Previous work on immunogenic outcomes from SARS-CoV-2 vaccination reveals that PLWH mounts overall similar serum IgG immunity to vaccination,[7,24,25,27,28] with serum IgG production and decay rates estimated via mechanistic modeling also found to be similar to HIV-negative individuals.[7] Where serum IgG spike and RBD features are the most populated feature type in our work (28 features in total), all of them are found to be the least-informative features for RF model classification (Figure 1F), with no median weights deviating from $\sim$0 during training (Figures 2D and 2E). Therefore, we find that the results of the RF approach are in agreement with previous literature for serum-based IgG features, with PLWH mounting indistinguishable IgG responses from an age-matched HIV-negative group. Serum IgG is primarily produced by B cells in the bone marrow, spleen, and lymph nodes.[41,42] Despite evidence for T cell dysregulations due to ART-suppressed HIV viremia, there is likely effective restoration of the systemic immunity of these central immune organs associated with mounting serum IgG responses to a degree where vaccine-elicited serum IgG responses are uninformative via an RF approach.

Gut-associated lymphoid tissue contains a distinct repertoire of lymphocytes that regulate mucosal humoral immunity[43]; further, plasmablasts responsible for IgA secretion are believed to migrate between bone marrow, blood, and mucus.[44] IgA is the first line of defense against SARS-CoV-2 infection at mucosal surfaces.[45,46] During the initial stages of HIV-1 infection, $CD4^{+}$ T cells in the mucosal immune system have been shown to be more significantly impacted than systemic $CD4^{+}$ T cells[47]; further, IgA production and maintenance is known to be dysregulated by HIV-1.[48] The extent to which mucosal immunity is restored, or further dysregulated, among PLWH on ART is not fully known.[49,50] In the context of live attenuated influenza vaccines in healthy young adults, mucosal and systemic humoral responses were found to be regulated by separate and distinct mechanisms.[51] Therefore, we were interested in determining whether an RF approach reveals a unique saliva-based immunogenic signature among HIV+ ART-suppressed individuals who received repeated SARS-CoV-2 vaccinations. In the dataset used in this work, the overall amplitude of saliva IgA responses both pre- and post-primary series remained relatively unchanged and was not determined to be a strong statistical differentiator between PLWH and the control group, while some significant statistical differences were reported for a subset of IgG.[7] However, our ablation analysis revealed that saliva IgA measures contribute to the minimally stratified combination of features, suggesting that saliva IgA is highly informative to differentiate the PLWH SARS-CoV-2 vaccine immunologic response from the HIV-negative control. This result may be partially explained by previous clinical literature. For example, PLWH exhibit chronic antigenic stimulation leading to B cell dysregulation and reduced IgA-producing plasmablasts,[52] where the proposed mechanism affecting IgA production is HIV nef protein penetrating B cells and blocking cytokine signaling.[53] Thus, our finding that saliva-based IgA as a key biomarker distinguishing the PLWH vaccine response highlights the utility of RF to detect subtle dysregulations in mucosal immune profiles not previously found by statistical or mechanistic modeling approaches.[7] Importantly, we did not design an experimental assay to evaluate this mechanism. Thus, this interpretation is speculative and should be regarded as hypothesis generating rather than confirmatory.

ML-generated synthetic data have incredible promise to increase diversity and robustness in medical and health-care datasets as well as making reproducibility of results more achievable through datasets that can be shared while preserving the privacy of protected health information.[54,55] The human immune system is incredibly complex, and the degree to which its myriad of components are functionally related is not well known.[56] ML techniques that can capture these underlying relationships are valuable for overcoming data limitations for training and model validation and reducing ethical constraints on data sharing. Furthermore, mechanistic modeling of the relationship between clinical outcomes and underlying immunological processes is often limited due to practical identifiability from data scarcity,[57]

particularly in longitudinal vaccine studies where frequent sampling and comprehensive immune profiling may not be feasible.[39,58–63] Therefore, synthetic data that preserve the local and global characteristics of the original dataset's structure would enable *in silico* testing of immunological hypotheses through computational approaches. We explore synthetic data generation using unsupervised methods such as GMM, MVN, and KNN as well as supervised methods such as SMOTE. Surprisingly, we find that SMOTE produces the least favorable outcome as quantified by KLD divergence, but the most favorable outcome by qualitative PCA comparison to the original data (Figure S8C versus Figure 1C) as well as quantitative comparison of feature importance behavior from RF model outcomes (Figure 4B). GMM achieves the lowest KLD (Figure S8A); however, it fails to preserve the data structure from PCAs (Figure S6A) and leads to inflated feature importance scores (Figure 4B). In a TSTR evaluation, an RF trained exclusively on cGMM synthetic data generalized to held-out real patients, closely matching a real-trained baseline (Figure 5). Feature-importance rankings were also preserved (Spearman's $\rho = 0.736$), indicating that our model trained entirely on synthetic data generalizes to real patient data while maintaining mechanistic interpretability under leakage-controlled conditions. If such generators can be extended to preserve longitudinal dependencies, they would enable *in silico* augmentation and validation of ODE-based non-linear mixed-effects (NLME) models via simulation-estimation workflows, improving parameter identifiability, power/sample-size planning, and robustness of inference without requiring additional patients.

Our results underscore how different approaches may capture various aspects of the global and local structure within the data and further show that different synthetic data generation approaches capture distinct aspects of the immunological response. A hybrid approach, combining the strength of GMM in modeling global distributions with SMOTE's ability to preserve local feature relationships, might better recapitulate the complex immune signatures observed in vaccine responses. Future work will explore such hybrid methodologies alongside advanced deep learning approaches, such as variational autoencoders (VAEs), generative adversarial networks (GANs), and diffusion- and energy-based models, to further enhance generative capabilities and improve model robustness. Further, our cohort is modest in size (68 PLWH, 23 controls) and predominantly older males, which constrains demographic breadth; consequently, generalizability is most direct to older, male, ART-suppressed PLWH, and extrapolation to broader populations should be made cautiously. Vaccine product and schedule (and calendar time) are plausible confounders of immunologic readouts. While our nested CV, imbalance-aware metrics, and TSTR analysis support robust model evaluation within this cohort, these findings should be interpreted within the context of an older predominantly male PLWH population; future work will evaluate generalizability in larger, demographically diverse cohorts with explicit vaccine-product stratification.

METHODS

Study approval

Vaccinations were not provided as a part of this study. All study participants provided informed written consent. The study protocol and consent form were approved by the University of Toronto Research Ethics Board (RIS #40713) and Sinai Health REB (21-0223-E). The study was conducted in accordance with the protocol, applicable regulations, and guidelines for good clinical practice (GCP), Health Canada's regulations, and the Tri-Council Policy Statement: Ethical Conduct for Research Involving Humans (TCPS 2.0).

Clinical data acquisition and description

This study used SARS-CoV-2 vaccination data for three doses of vaccine previously published in Matveev et al.[7] Primary booster series data, corresponding to visits 1 through 9, were acquired for this work through a data sharing agreement between authors J.M.H. and M.O. Visit acquisition times can be found in the timeline schematic in Figure 1A. Briefly, visits 1–9 occurred over 48 weeks post-dose 1 vaccination and consisted of individuals who received three doses of SARS-CoV-2 vaccination. These data comprise 91 individuals: 23 HIV− and 63 HIV+. Individuals were predominantly male, recruited from a clinic with a large MSM population. Visits 10 and 11 IgG spike and RBD serum data corresponding to post-SARS-CoV-2 vaccinations 4 and 5, respectively, were also used in this work. These data were collected by the A.-C.G. lab in a follow-up study to Matveev et al.[7] (see acknowledgments) and are unpublished primary data. The timeline of study protocol can be found in Figure 1B. We enrolled PLWH under active clinical care in the greater Toronto region during periods when public health guidance in North America and the United Kingdom recommended additional COVID-19 booster doses for older adults and for persons with moderate/severe immunocompromise; consequently, some participants had up to five total doses (primary series plus additional/seasonal boosters). Among PLWH, the range of time of viral load suppression is 5–30 years (min/max), with median and mean years of viral load suppression of 18.4 and 19, respectively. A brief description of each biomarker can be found in Table S2.

Antibody detection in serum

Antibody detection in serum for visits 10 and 11 were carried out as described in Matveev et al.[7] An automated ELISA was employed to measure total IgG antibody levels against the full-length spike trimer, RBD, and nucleocapsid, as described previously.[64,65] In summary, 384-well microplates were precoated with spike (SmT1), RBD, or nucleocapsid antigens provided by the National Research Council of Canada (NRC). Key steps included blocking with BLOTTO (Thermo Fisher Scientific) and incubation with serum dilutions of 1:160 or 1:2,560, followed by incubation with HRP-conjugated human anti-IgG (IgG#5, supplied by NRC). Detection was carried out using ELISA Pico Chemiluminescent Substrate (Thermo Fisher Scientific), and chemiluminescence was measured with an EnVision 2105 Multimode Plate Reader (PerkinElmer). Raw chemiluminescence values were normalized using a synthetic standard included on each plate (VHH72-Fc from NRC for spike/RBD or anti-N IgG from Genscript, #A02039). These values were further converted to BAU/mL using the WHO International Standard 20/136 as a calibrator.[64] Seropositivity thresholds for the 1:160 dilution were established based on 3 standard deviations above the mean of control samples.[64]

Missing data imputation

Clinical features with missing entries are common when dealing with immunological clinical data,[66] with the imputation necessary to carry out basic ML algorithms.[67] We imputed missing data using the R library `missForest`[68] (version 1.5) to each class (HIV− and HIV+) separately. missForest works by iteratively training an RF model on the observed data to predict and replace missing values for each feature, cycling through all features until convergence. Imputation of clinical data using missForest has been widely implemented and shown to handle mix-type data well.[69–71] In this work, all 63 of our clinical features were continuous variables. Normalized root-mean-squared error (NRMSE) was computed to assess imputation accuracy. The NRMSE for the HIV− and HIV+ imputed values were calculated to be 0.38 and 0.36, respectively. The most commonly imputed feature types were the saliva antibody features. In order to determine the accuracy of imputed values, in the supplemental information we assess the influence of low frequency of mutual missing values on imputed results by randomly removing observed values from all feature compartments and imputing them using missForest to measure the model accuracy. The NRMSE falls within a reasonable range for missForest's performance as a function of increasing percentage of missingness (Figure S9), and the mean and variance of all biomarkers remain stable across increasing levels of missingness (Figures S10 and S11, respectively).

Correlation network visualization

We visualized the correlation network structure of the clinical features, with each feature represented as a node and with the network layout calculated using the t-SNE algorithm[34] applied to the adjacency matrix. It is recommended that t-SNE perplexity satisfy $3 \times \text{perplexity} < \text{feature count} - 1$; therefore, we set the perplexity to $(63 - 1)/3$, where 63 is the number of features. The network node size was proportional to RF feature importance. Vertex color-code varied and is described in the respective figure caption. For visualization purposes, we plotted an edge between a pair of features only if the correlation was statistically significant (p values < 0.05). The t-SNE layout was implemented using the *Rt-SNE* function from the `Rt-SNE` library in R. To visualize and validate t-SNE networks, we also employed UMAP in R with the *umap* function in the R library `uwot`.

Model development and model performance metrics

PCA is an unsupervised dimensionality reduction technique that transforms high-dimensional data into orthogonal components that preserve as much variance as possible. We performed PCA on the full dataset to validate whether an unsupervised clustering technique leads to separate clusters composed of our data classes (HIV+ and HIV−). PCA was implemented using the R function *prcomp* in the `stats` library, with confidence ellipses of levels of 1 standard deviation drawn using the *dataEllipse* function in the `car` library. LDA was used to provide initial insights into class separability. LDA was implemented using the *lda* function in the `MASS` library in R.

We employed the RF algorithm,[72] a non-linear ensemble-based method, to quantify the complex non-linear relationships between HIV+ and HIV− immunological responses. All training sets were randomly downsampled to balanced population sizes of 20:20 for HIV−:HIV+ individuals. The number of trees was initially increased until the ORR error stabilized and was then fixed throughout this work to a value of 200. Random holdouts of population proportions of 3:12 (HIV−:HIV+) were excluded from training and used to assess model performance. 1,000 stochastically generated instances of the RF algorithm (each with randomly selected training and testing sets) was considered to assess RF model performance. Majority tree visualization was explored to "look under the hood" of every given RF-training and RF-testing landscape to assess how features were being weighted for each randomly sampled training and holdout (test) set. To do this, we constructed a model explanation function using the *lime* and *explain* functions from the `lime` library in R. Feature weights from every RF model were then stored and visualized (see supplemental information for individual model examples). For all RF models, a threshold of 0.5 was used. The repercussion of a 0.5 threshold on individual prediction accuracy with a 0.5 threshold is explored in the supplemental information. Classifiers were evaluated with nested cross-validation: an outer 5-fold CV (with repeats) for unbiased performance estimation and an inner 5-fold CV for hyperparameter selection. For RFs, the inner loop tuned *mtry*, minimal node size, sample fraction, and sampling with/without replacement via a grid search. For TSTR, all preprocessing (standardization, PCA, cGMM fitting) was done strictly inside each training fold and applied those transforms to the test fold only. We additionally ran 1,000 random label-permutation controls (chance-level AUC) and feature-ablation analyses to assess overfitting and stability.

An underlying assumption of the RF algorithm is the statistical independence of all observations. In this study, while the patients are independent, the samples collected from various vaccines for the same patient are not. To address this, we ensured that samples from the same patient taken during multiple vaccine trials remained within the same fold to prevent them from being dispersed across both training and test datasets. Furthermore, we implemented a two-layer cross-validation procedure designed to optimize model hyperparameters while ensuring that predictions were made exclusively on samples not seen during the training process to guarantee complete independence from intra-patient correlations.

Feature importance

Gini Impurity measures the likelihood of incorrect classification of a randomly chosen element if it were labeled according to the class distribution in a dataset. RF splits the dataset at each node using a feature that minimizes Gini Impurity. At each split, the Gini Impurity before and after the split is calculated, and the reduction in Gini Impurity due to that split is recorded. Feature importance in RF is derived by summing the total reduction in Gini Impurity across all trees in the forest, weighted by the number of samples passing through each node. This was implemented using the *varImp* function in R. The cumulative average from each importance calculation was then calculated across all RF models.

Ablation analysis

To assess the sensitivity of RF performance on our features and, further, to identify and select the most important features that contributed the most to the model accuracy and performance,

we implemented two types of ablation analysis. Reverse ablation, whereby features are removed as a function of most importance until the two least-important features remain, was conducted to determine the minimal accuracy of our two least-important features. Forward ablation, which begins with the two most important features and then iteratively adds in features as a function of their importance, was conducted to determine the subset of features that lead to equivalent model performance.

For every ablation index, the RF model was employed as described above with 1,000 stochastically generated instances of training and test sets. Equivalent model performance was assessed by computing a Wilcoxon test between the resulting distributions of the AUC-ROC and F1 scores and their respective "full dataset" counterparts for the forward ablation algorithm. p values below 0.05 thus indicate non-equivalent model performance, whereas p values above 0.05 indicate equivalent model performance to the full 63 feature dataset. Where p values cross from below to above 0.05, therefore, suggests the minimal subset of features to achieve equivalent model performance when using the full 63 feature dataset.

Simulated data

A major motivation for this study was to develop a robust framework for generating synthetic immunological data that assimilates the complex patterns observed in our original vaccine response dataset. We implemented six synthetic data generating techniques: MVN, GMM, SMOTE, and KNN with $k = 3$, $k = 10$ (determined as the optimal k value), and $k = 20$ ($2\times$ optimal). For unsupervised techniques, HIV− and HIV+ data were generated separately from one another. All synthetic datasets were generated equal to our original dataset's class balance and feature size: each was implemented to uniquely generate 91 individuals (23 HIV− and 68 HIV+) as well as 63 corresponding immunological features. SMOTE is a supervised technique typically used to generate synthetic data to achieve a balanced dataset. Here, we applied SMOTE to generate synthetic samples for both the minority and the majority classes. MVN, GMM, SMOTE, and KNN are all implemented in R using the `MASS`, `mclust`, `smotefamily`, and `FNN` libraries, respectively.

Evaluation of simulated data approaches

We used KLD to evaluate and rank the synthetic data methods by measuring how much the original data are different from the synthetic dataset that we generated via the various methods described above. The KLD is calculated using the *KLD* function in the `LaplacesDemon` library in R. We calculated the KLD between all 63 synthetic and original features. The mean KLD was then computed by taking the mean of all the KLD for each synthetic approach; similarly, we also computed the total variance across all KLD for each synthetic approach. Synthetic approaches were then ranked by their mean and total variance KLDs: a low mean KLD, computed across all features, suggests that the synthetic data approach is close to the original distribution of features, while a low total KLD variance means that the KLD values across features being synthetically produced contain a consistent level of similarity to the original data. Low total KLD variance also suggests that there are fewer subsets of features deviating significantly from the original data. Finally, for the best-ranked synthetic method, we computed variance in KLD as a function of feature type to assess if certain synthetic features were closer to the original data than others.

We also utilized PCA to assess how similar synthetic data features were to the original data. To do this, we performed PCA on the original data and then projected each synthetic dataset onto the PCA space of the original dataset. Ellipses drawn to the first standard deviations of the resulting first two principal components were then used to visually inspect clustering behavior. If the ellipses representing the synthetic data generated by one method overlapped significantly with the ellipses from the original data in the PCA space, it suggested that the synthetic data closely resembled the distribution and structure of the original data in the reduced dimensionality space.

Conditional GMM synthesis and TSTR

For each stratified k-fold, we held out a real test fold and fit cGMMs on the training fold only (test fold never used for scaling, PCA, or model fitting). Training features were standardized by fold-specific means/SDs and projected to a PCA subspace retaining ≥95% variance (dimension capped at $min(p, n_{minclass} - 1)$ for stability). For each class, a GMM was fit in PC; the BIC-minimizing model was chosen. We sampled per-class synthetic points (size ≈10× the real training fold) and then back-projected to the original feature space. An RF was trained only on the synthetic set and evaluated on the held-out real fold (TSTR). As a baseline, an RF trained on the real training fold was evaluated on the same test fold. Performance metrics were ROC AUC, PR AUC, accuracy, precision, recall, and F_1; mechanistic concordance was quantified by Spearman rank correlation between RF importance vectors (synthetic trained versus real trained). Analyses were performed in R using `mclust`, `MASS`, `ranger`, `pROC`, and `PRROC` libraries with fixed seeds for reproducibility.

RESOURCE AVAILABILITY

Lead contact

Requests for further information should be directed to the lead contact, Chapin S. Korosec (chapinskorosec@gmail.com).

Materials availability

No unique reagents were generated in the study.

Data and code availability

The human data from doses 1–3, along with the synthetic data and code used in this work, are available in a public GitHub repository, accessible here or via the fig share https://doi.org/10.6084/m9.figshare.30593918.[73] For access to the dose 4 and dose 5 human data, please contact M.O.

ACKNOWLEDGMENTS

This project is supported by the NRC-Fields Mathematical Sciences Collaboration Centre and the AI4PH program, sponsored by the Dalla Lana School of Public Health, University of Toronto. C.S.K. acknowledges NSERC postdoctoral funding. J.M.H. acknowledges funding from NSERC, CIHR, NSERC EIDM, and the York Research Chair Program. J.M.C. acknowledges the support of the National Institutes of Health (grant nos. R21-AI143443-01A1 and R01-OD011095). The authors are thankful to the Anne-Claude Gingras group (Lunenfeld-Tanenbaum Research Institute, Sinai Health, Toronto, Canada) for the IgG ELISA on sera from the two final study visits.

AUTHOR CONTRIBUTIONS

Conceptualization, C.S.K., J.M.C., M.S.G., and J.M.H.; funding acquisition, C.S.K., M.O., and J.M.H.; data curation, C.S.K. and V.A.M.; visualization, C.S.K. and M.S.G.; methodology, C.S.K., J.M.C., J.M.H., and M.S.G.; investigation, all authors; validation, C.S.K. and V.A.M.; formal analysis, C.S.K. and M.S.G.; supervision, M.S.G., J.M.H., and J.M.C.; writing – original draft, C.S.K.; preparation, C.S.K.

DECLARATION OF INTERESTS

The authors declare no competing interests.

SUPPLEMENTAL INFORMATION

Supplemental information can be found online at https://doi.org/10.1016/j.patter.2025.101474.

Received: June 13, 2025
Revised: October 18, 2025
Accepted: December 10, 2025
Published: March 4, 2026

REFERENCES

1. Bekker, L.-G., Beyrer, C., Mgodi, N., Lewin, S.R., Delany-Moretlwe, S., Taiwo, B., Masters, M.C., and Lazarus, J.V. (2023). HIV infection. Nat. Rev. Dis. Primers *9*, 42. https://doi.org/10.1038/s41572-023-00452-3.
2. Carter, A., Zhang, M., Tram, K.H., Walters, M.K., Jahagirdar, D., Brewer, E.D., Novotney, A., Lasher, D., Mpolya, E.A., and Vongpradith, A. (2024). Global, regional, and national burden of HIV/AIDS, 1990–2021, and forecasts to 2050, for 204 countries and territories: the Global Burden of Disease Study 2021. The Lancet HIV *11*, e807–e822. https://doi.org/10.1016/S2352-3018(24)00212-1.
3. Teeraananchai, S., Kerr, S.J., Amin, J., Ruxrungtham, K., and Law, M.G. (2017). Life expectancy of HIV-positive people after starting combination antiretroviral therapy: a meta-analysis. HIV Med. *18*, 256–266. https://doi.org/10.1111/hiv.12421.
4. Deeks, S.G., Overbaugh, J., Phillips, A., and Buchbinder, S. (2015). HIV infection. Nat. Rev. Dis. Primers *1*, 15035. https://doi.org/10.1038/nrdp.2015.35.
5. Bono, V., Augello, M., Tincati, C., and Marchetti, G. (2022). Failure of CD4+ T-cell Recovery upon Virally-Effective cART: an Enduring Gap in the Understanding of HIV+ Immunological non-Responders. New Microbiol. *45*, 155–172.
6. Lv, T., Cao, W., and Li, T. (2021). HIV-Related Immune Activation and Inflammation: Current Understanding and Strategies. J. Immunol. Res. *2021*, 7316456. https://doi.org/10.1155/2021/7316456.
7. Matveev, V.A., Mihelic, E.Z., Benko, E., Budylowski, P., Grocott, S., Lee, T., Korosec, C.S., Colwill, K., Stephenson, H., Law, R., et al. (2023). Immunogenicity of COVID-19 vaccines and their effect on HIV reservoir in older people with HIV. iScience *26*, 107915. https://doi.org/10.1016/J.ISCI.2023.107915.
8. Saout, C.L., Lane, H.C., and Catalfamo, M. (2012). The role of cytokines in the pathogenesis and treatment of HIV infection. Cytokine Growth Factor Rev. *23*, 207–214. https://doi.org/10.1016/j.cytogfr.2012.05.007.
9. Höft, M.A., Burgers, W.A., and Riou, C. (2024). The immune response to SARS-CoV-2 in people with HIV. Cell. Mol. Immunol. *21*, 184–196. https://doi.org/10.1038/s41423-023-01087-w.
10. Burton, D.R. (2002). Antibodies, viruses and vaccines. Nat. Rev. Immunol. *2*, 706–713.
11. Levy, I., Wieder-Finesod, A., Litchevsky, V., Biber, A., Indenbaum, V., Olmer, L., Huppert, A., Mor, O., Goldstein, M., Levin, E.G., et al. (2021). Immunogenicity and safety of the BNT162b2 mRNA COVID-19 vaccine in people living with HIV-1. Clin. Microbiol. Infect. *27*, 1851–1855. https://doi.org/10.1016/j.cmi.2021.07.031.
12. Pai, N.P., Karellis, A., Kim, J., and Peter, T. (2020). Modern diagnostic technologies for HIV. Lancet. HIV *7*, e574–e581. https://doi.org/10.1016/S2352-3018(20)30190-9.
13. Xiang, Y., Du, J., Fujimoto, K., Li, F., Schneider, J., and Tao, C. (2022). Application of artificial intelligence and machine learning for HIV prevention interventions. Lancet. HIV *9*, e54–e62.
14. Lee, E.K., Nakaya, H.I., Yuan, F., Querec, T.D., Burel, G., Pietz, F.H., Benecke, B.A., and Pulendran, B. (2016). Machine Learning for Predicting Vaccine Immunogenicity. Interfaces *46*, 368–390.
15. Kernéis, S., Launay, O., Turbelin, C., Batteux, F., Hanslik, T., and Boëlle, P.Y. (2014). Long-term immune responses to vaccination in HIV-infected patients: A systematic review and meta-analysis. Clin. Infect. Dis. *58*, 1130–1139. https://doi.org/10.1093/cid/cit937.
16. El Chaer, F., and El Sahly, H.M. (2019). Vaccination in the Adult Patient Infected with HIV: A Review of Vaccine Efficacy and Immunogenicity. Am. J. Med. *132*, 437–446. https://doi.org/10.1016/j.amjmed.2018.12.011.
17. Geretti, A.M., and Doyle, T. (2010). Immunization for HIV-positive individuals. Curr. Opin. Infect. Dis. *23*, 32–38.
18. García, F., Climent, N., Guardo, A.C., Gil, C., León, A., Autran, B., Lifson, J.D., Martínez-Picado, J., Dalmau, J., Clotet, B., et al. (2013). A dendritic cell-based vaccine elicits T cell responses associated with control of HIV-1 replication. Sci. Transl. Med. *5*, 166ra2. https://doi.org/10.1126/scitranslmed.3004682.
19. Niessl, J., Baxter, A.E., Mendoza, P., Jankovic, M., Cohen, Y.Z., Butler, A.L., Lu, C.L., Dubé, M., Shimeliovich, I., Gruell, H., et al. (2020). Combination anti-HIV-1 antibody therapy is associated with increased virus-specific T cell immunity. Nat. Med. *26*, 222–227. https://doi.org/10.1038/s41591-019-0747-1.
20. Pollard, A.J., and Bijker, E.M. (2021). A guide to vaccinology: from basic principles to new developments. Nat. Rev. Immunol. *21*, 83–100. https://doi.org/10.1038/s41577-020-00479-7.
21. Cox, R.J., and Brokstad, K.A. (2020). Not just antibodies: B cells and T cells mediate immunity to COVID-19. Nat. Rev. Immunol. *20*, 581–582. https://doi.org/10.1038/s41577-020-00436-4.
22. Brodin, P., and Davis, M.M. (2017). Human immune system variation. Nat. Rev. Immunol. *17*, 21–29. https://doi.org/10.1038/nri.2016.125.
23. Alirezaylavasani, A., Skeie, L.G., Egner, I.M., Chopra, A., Dahl, T.B., Prebensen, C., Vaage, J.T., Halvorsen, B., Lund-Johansen, F., Tonby, K., et al. (2024). Vaccine responses and hybrid immunity in people living with HIV after SARS-CoV-2 breakthrough infections. npj Vaccines *9*, 185. https://doi.org/10.1038/s41541-024-00972-3.
24. Frater, J., Ewer, K.J., Ogbe, A., Pace, M., Adele, S., Adland, E., Alagaratnam, J., Aley, P.K., Ali, M., Ansari, M.A., et al. (2021). Safety and immunogenicity of the ChAdOx1 nCoV-19 (AZD1222) vaccine against SARS-CoV-2 in HIV infection: a single-arm substudy of a phase 2/3 clinical trial. Lancet. HIV *8*, e474–e485. https://doi.org/10.1016/S2352-3018(21)00103-X.
25. Brumme, Z.L., Mwimanzi, F., Lapointe, H.R., Cheung, P.K., Sang, Y., Duncan, M.C., Yaseen, F., Agafitei, O., Ennis, S., Ng, K., et al. (2022). Humoral immune responses to COVID-19 vaccination in people living with HIV receiving suppressive antiretroviral therapy. npj Vaccines *7*, 28. https://doi.org/10.1038/s41541-022-00452-6.
26. Vergori, A., Cozzi Lepri, A., Cicalini, S., Matusali, G., Bordoni, V., Lanini, S., Meschi, S., Iannazzo, R., Mazzotta, V., Colavita, F., et al. (2022). Immunogenicity to COVID-19 mRNA vaccine third dose in people living with HIV. Nat. Commun. *13*, 4922. https://doi.org/10.1038/s41467-022-32263-7.
27. Noe, S., Ochana, N., Wiese, C., Schabaz, F., Von Krosigk, A., Heldwein, S., Rasshofer, R., Wolf, E., and Jonsson-Oldenbuettel, C. (2022). Humoral response to SARS-CoV-2 vaccines in people living with HIV. Infection *50*, 617–623. https://doi.org/10.1007/s15010-021-01721-7.
28. Knudsen, M.L., Nielsen, S.D., and Heftdal, L.D. (2024). Immune responses to mRNA-based vaccines given as a third COVID-19 vaccine dose in

people living with HIV—a literature review. Apmis *132*, 236–244. https://doi.org/10.1111/apm.13379.

29. Augello, M., Bono, V., Rovito, R., Tavelli, A., Santoro, A., Tincati, C., Vergori, A., Azzini, A.M., Righi, E., Spiteri, G., et al. (2025). Long-term immune responses to SARS-CoV-2 Omicron BA.4/5 mRNA booster in people living with HIV. Commun. Med. *5*, 92. https://doi.org/10.1038/s43856-025-00799-6.
30. Cascarano, A., Mur-Petit, J., Hernández-González, J., Camacho, M., de Toro Eadie, N., Gkontra, P., Chadeau-Hyam, M., Vitrià, J., and Lekadir, K. (2023). Machine and deep learning for longitudinal biomedical data: a review of methods and applications. Artif. Intell. Rev. *56*, 1711–1771. https://doi.org/10.1007/s10462-023-10561-w.
31. Goronzy, J.J., and Weyand, C.M. (2013). Understanding immunosenescence to improve responses to vaccines. Nat. Immunol. *14*, 428–436. https://doi.org/10.1038/ni.2588.
32. Denisko, D., and Hoffman, M.M. (2018). Classification and interaction in random forests. Proc. Natl. Acad. Sci. USA *115*, 1690–1692.
33. Kumar, S.S., and Shaikh, T. (2017). Empirical evaluation of the performance of feature selection approaches on random forest. In 2017 international conference on computer and applications (ICCA) (IEEE), pp. 227–231.
34. Van der Maaten, L., and Hinton, G. (2008). Visualizing data using t-SNE. Journal of machine learning research *9*.
35. Tuv, E., Borisov, A., Runger, G., and Torkkola, K. (2009). Feature selection with ensembles, artificial variables, and redundancy elimination. J. Mach. Learn. Res. *10*, 1341–1366.
36. Halladin-Dabrowska, A., Kania, A., and Kopeć, D. (2020). The t-SNE algorithm as a tool to improve the quality of reference data used in accurate mapping of heterogeneous non-forest vegetation. Remote Sens. *12*, 39. https://doi.org/10.3390/RS12010039.
37. Moss, P. (2022). The T cell immune response against SARS-CoV-2. Nat. Immunol. *23*, 186–193. https://doi.org/10.1038/s41590-021-01122-w.
38. Samaan, P., Korosec, C.S., Budylowski, P., Chau, S.L., Pasculescu, A., Qi, F., Delgado-Brand, M., Tulunay, T.R., Mailhot, G., Dayam Roya, M., et al. (2025). mRNA vaccine-induced SARS-CoV-2 spike-specific IFN-γ and IL-2 T-cell responses are predictive of serological neutralization and are transiently enhanced by pre-existing cross-reactive immunity. J. Virol. *99*, e01685. https://doi.org/10.1128/jvi.01685-24.
39. Lin, J., Law, R., Korosec, C.S., Zhou, C., Koh, W.H., Ghaemi, M.S., Samaan, P., Ooi, H.K., Matveev, V., Yue, F., et al. (2022). Longitudinal Assessment of SARS-CoV-2-Specific T Cell Cytokine-Producing Responses for 1 Year Reveals Persistence of Multicytokine Proliferative Responses, with Greater Immunity Associated with Disease Severity. J. Virol. *96*, e0050922. https://doi.org/10.1128/jvi.00509-22.
40. Baduashvili, A., Guyatt, G., and Evans, A.T. (2019). ROC Anatomy—Getting the Most Out of Your Diagnostic Test. J. Gen. Intern. Med. *34*, 1892–1898. https://doi.org/10.1007/s11606-019-05125-0.
41. Young, C., and Brink, R. (2021). The unique biology of germinal center B cells. Immunity *54*, 1652–1664. https://doi.org/10.1016/j.immuni.2021.07.015.
42. Good-Jacobson, K.L., and Shlomchik, M.J. (2010). Plasticity and Heterogeneity in the Generation of Memory B Cells and Long-Lived Plasma Cells: The Influence of Germinal Center Interactions and Dynamics. J. Immunol. *185*, 3117–3125. https://doi.org/10.4049/jimmunol.1001155.
43. Janeway, C., Travers, P., Walport, M., and Shlomchik, M. (2001). Immunobiology: The Immune System in Health and Disease, *2* (New York: Garland Pub).
44. Mei, H.E., Yoshida, T., Sime, W., Hiepe, F., Thiele, K., Manz, R.A., Radbruch, A., and Dörner, T. (2009). Blood-borne human plasma cells in steady state are derived from mucosal immune responses. Blood *113*, 2461–2469. https://doi.org/10.1182/blood-2008-04-153544.
45. Wang, Z., Lorenzi, J.C.C., Muecksch, F., Finkin, S., Viant, C., Gaebler, C., Cipolla, M., Hoffmann, H.-H., Oliveira, T.Y., Oren, D.A., et al. (2021). Enhanced SARS-CoV-2 neutralization by dimeric IgA. Sci. Transl. Med. *13*, eabf1555. https://doi.org/10.1126/scitranslmed.abf1555.
46. Sheikh-Mohamed, S., Isho, B., Chao, G.Y.C., Zuo, M., Cohen, C., Lustig, Y., Nahass, G.R., Salomon-Shulman, R.E., Blacker, G., Fazel-Zarandi, M., et al. (2022). Systemic and mucosal IgA responses are variably induced in response to SARS-CoV-2 mRNA vaccination and are associated with protection against subsequent infection. Mucosal Immunol. *15*, 799–808. https://doi.org/10.1038/s41385-022-00511-0.
47. Hel, Z., McGhee, J.R., and Mestecky, J. (2006). HIV infection: first battle decides the war. Trends Immunol. *27*, 274–281. https://doi.org/10.1016/j.it.2006.04.007.
48. Xu, W., Santini, P.A., Sullivan, J.S., He, B., Shan, M., Ball, S.C., Dyer, W.B., Ketas, T.J., Chadburn, A., Cohen-Gould, L., et al. (2009). HIV-1 evades virus-specific IgG2 and IgA responses by targeting systemic and intestinal B cells via long-range intercellular conduits. Nat. Immunol. *10*, 1008–1017. https://doi.org/10.1038/ni.1753.
49. Allers, K., Puyskens, A., Epple, H.J., Schürmann, D., Hofmann, J., Moos, V., and Schneider, T. (2016). The effect of timing of antiretroviral therapy on CD4+ T-cell reconstitution in the intestine of HIV-infected patients. Mucosal Immunol. *9*, 265–274. https://doi.org/10.1038/mi.2015.58.
50. Tincati, C., Douek, D.C., and Marchetti, G. (2016). Gut barrier structure, mucosal immunity and intestinal microbiota in the pathogenesis and treatment of HIV infection. AIDS Res. Ther. *13*, 19. https://doi.org/10.1186/s12981-016-0103-1.
51. Thwaites, R.S., Uruchurtu, A.S.S., Negri, V.A., Cole, M.E., Singh, N., Poshai, N., Jackson, D., Hoschler, K., Baker, T., Scott, I.C., et al. (2023). Early mucosal events promote distinct mucosal and systemic antibody responses to live attenuated influenza vaccine. Nat. Commun. *14*, 8053. https://doi.org/10.1038/s41467-023-43842-7.
52. Buckner, C.M., Moir, S., Ho, J., Wang, W., Posada, J.G., Kardava, L., Funk, E.K., Nelson, A.K., Li, Y., Chun, T.-W., and Fauci, A.S. (2013). Characterization of Plasmablasts in the Blood of HIV-Infected Viremic Individuals: Evidence for Nonspecific Immune Activation. J. Virol. *87*, 5800–5811. https://doi.org/10.1128/jvi.00094-13.
53. Qiao, X., He, B., Chiu, A., Knowles, D.M., Chadburn, A., and Cerutti, A. (2006). Human immunodeficiency virus 1 Nef suppresses CD40-dependent immunoglobulin class switching in bystander B cells. Nat. Immunol. *7*, 302–310. https://doi.org/10.1038/ni1302.
54. Chen, R.J., Lu, M.Y., Chen, T.Y., Williamson, D.F.K., and Mahmood, F. (2021). Synthetic data in machine learning for medicine and healthcare. Nat. Biomed. Eng. *5*, 493–497. https://doi.org/10.1038/s41551-021-00751-8.
55. Giuffrè, M., and Shung, D.L. (2023). Harnessing the power of synthetic data in healthcare: innovation, application, and privacy. npj Digit. Med. *6*, 1–8. https://doi.org/10.1038/s41746-023-00927-3.
56. Iwasaki, A., and Medzhitov, R. (2015). Control of adaptive immunity by the innate immune system. Nat. Immunol. *16*, 343–353. https://doi.org/10.1038/ni.3123.
57. Korosec, C.S., Betti, M.I., Dick, D.W., Ooi, H.K., Moyles, I.R., Wahl, L.M., and Heffernan, J.M. (2023). Multiple cohort study of hospitalized SARS-CoV-2 in-host infection dynamics: Parameter estimates, identifiability, sensitivity and the eclipse phase profile. J. Theor. Biol. *564*, 111449. https://doi.org/10.1016/j.jtbi.2023.111449.
58. Farhang-sardroodi, S., Korosec, C.S., Gholami, S., Craig, M., Moyles, I.R., Ghaemi, M.S., Ooi, H.K., and Heffernan, J.M. (2021). Analysis of Host Immunological Response of Adenovirus-Based COVID-19 Vaccines. Vaccines *9*, 861.
59. Moyles, I.R., Korosec, C.S., and Heffernan, J.M. (2023). Determination of significant immunological timescales from mRNA-LNP-based vaccines in humans. J. Math. Biol. *86*, 86. https://doi.org/10.1007/s00285-023-01919-3.
60. Korosec, C.S., Farhang-Sardroodi, S., Dick, D.W., Gholami, S., Ghaemi, M.S., Moyles, I.R., Craig, M., Ooi, H.K., and Heffernan, J.M. (2022). Long-term durability of immune responses to the BNT162b2 and

mRNA-1273 vaccines based on dosage, age and sex. Sci. Rep. *12*, 21232. https://doi.org/10.1038/s41598-022-25134-0.

61. Korosec, C.S., Dick, D.W., Moyles, I.R., and Watmough, J. (2024). SARS-CoV-2 booster vaccine dose significantly extends humoral immune response half-life beyond the primary series. Sci. Rep. *14*, 8426. https://doi.org/10.1038/s41598-024-58811-3.

62. Gholami, S., Korosec, C.S., Farhang-Sardroodi, S., Dick, D.W., Craig, M., Ghaemi, M.S., Ooi, H.K., and Heffernan, J.M. (2023). A mathematical model of protein subunits COVID-19 vaccines. Math. Biosci. *358*, 108970. https://doi.org/10.1016/J.MBS.2023.108970.

63. Banuet-Martinez, M., Yang, Y., Jafari, B., Kaur, A., Butt, Z.A., Chen, H.H., Yanushkevich, S., Moyles, I.R., Heffernan, J.M., and Korosec, C.S. (2023). Monkeypox: A review of epidemiological modelling studies and how modelling has led to mechanistic insight. Epidemiol. Infect. *151*, E121. https://doi.org/10.1017/S0950268823000791.

64. Colwill, K., Galipeau, Y., Stuible, M., Gervais, C., Arnold, C., Rathod, B., Abe, K.T., Wang, J.H., Pasculescu, A., Maltseva, M., et al. (2022). A scalable serology solution for profiling humoral immune responses to SARS-CoV-2 infection and vaccination. Clin. Transl. Immunology *11*, e1380. https://doi.org/10.1002/cti2.1380.

65. Isho, B., Abe, K.T., Zuo, M., Jamal, A.J., Rathod, B., Wang, J.H., Li, Z., Chao, G., Rojas, O.L., Bang, Y.M., et al. (2020). Persistence of serum and saliva antibody responses to SARS-CoV-2 spike antigens in COVID-19 patients. Sci. Immunol. *5*, eabe5511. https://doi.org/10.1126/sciimmunol.abe5511.

66. Liang, W., Liang, H., Ou, L., Chen, B., Chen, A., Li, C., Li, Y., Guan, W., Sang, L., Lu, J., et al. (2020). Development and validation of a clinical risk score to predict the occurrence of critical illness in hospitalized patients with COVID-19. JAMA Intern. Med. *180*, 1081–1089. https://doi.org/10.1001/jamainternmed.2020.2033.

67. Austin, P.C., White, I.R., Lee, D.S., and van Buuren, S. (2021). Missing Data in Clinical Research: A Tutorial on Multiple Imputation. Can. J. Cardiol. *37*, 1322–1331. https://doi.org/10.1016/j.cjca.2020.11.010.

68. Stekhoven, D.J., and Bühlmann, P. (2012). Missforest-Non-parametric missing value imputation for mixed-type data. Bioinformatics *28*, 112–118. https://doi.org/10.1093/bioinformatics/btr597.

69. Waljee, A.K., Mukherjee, A., Singal, A.G., Zhang, Y., Warren, J., Balis, U., Marrero, J., Zhu, J., and Higgins, P.D. (2013). Comparison of imputation methods for missing laboratory data in medicine. BMJ Open *3*, e002847. https://doi.org/10.1136/bmjopen-2013-002847.

70. Khademi, A. (2020). Flexible imputation of missing data 2nd edition. J. Stat. Softw. *93*, 1–4. https://doi.org/10.18637/jss.v093.b01.

71. Tiwaskar, S., Rashid, M., and Gokhale, P. (2024). Impact of machine learning-based imputation techniques on medical datasets- a comparative analysis. Multimed. Tools Appl. *84*, 5905–5925. https://doi.org/10.1007/s11042-024-19103-0.

72. Breiman, L. (2001). Random Forests. Mach. Learn. *45*, 5–32. https://doi.org/10.1023/A:1010933404324.

73. Korosec, C. S. (2025). Code and data for rf patterns paper (version 1.0.0). Figshare. doi:https://doi.org/10.6084/m9.figshare.30593918 software.

Patterns, Volume *7*

Supplemental information

Modeling of longitudinal immune profiles reveals distinct immunogenic signatures following five COVID-19 vaccinations among people living with HIV

Chapin S. Korosec, Jessica M. Conway, Vitaliy A. Matveev, Mario Ostrowski, Jane M. Heffernan, and Mohammad Sajjad Ghaemi

Supplementary Material: Modelling of longitudinal immune profiles reveals distinct immunogenic signatures following five COVID-19 vaccinations among people with HIV

Chapin S. Korosec[1,2,3], Jessica M. Conway[4], Vitaliy A. Matveev[5], Mario Ostrowski[5,6,7], Jane M. Heffernan[2,3], Mohammad Sajjad Ghaemi[8]

[1]Department of Mathematics and Statistics, University of Guelph, Guelph, ON, Canada.[2]Modelling Infection and Immunity Lab, Mathematics and Statistics, York University, Toronto, ON, Canada.[3]Centre for Disease Modelling, Mathematics and Statistics, York University, Toronto, ON, Canada.[4]Department of Mathematics, Pennsylvania State University, University Park, Pennsylvania, United States of America.[5]Department of Medicine, University of Toronto, Toronto, ON, Canada.[6]Department of Immunology, University of Toronto, Toronto, ON, Canada.[7]Keenan Research Centre for Biomedical Science, St. Michael's Hospital, Unity Health, Toronto, ON, Canada.[8]Digital Technologies Research Centre, National Research Council Canada, Toronto, ON, Canada.

*Corresponding authors: chapinSkorosec@gmail.com, jmheffer@yorku.ca, mohammadsajjad.ghaemi@nrc-cnrc.gc.ca

Contents

S1 ML benchmark analysis **3**

S2 Additional detail on biomarker descriptions **4**

S3 PWH viral load metadata **5**

S4 RF classification probabilities **8**

S4.1 Synthetic Data Analysis 13

S5 Imputation analysis **16**

S6 UMAP to compliment tSNE **19**

S1 ML benchmark analysis

**Method**	**Accuracy**	**ROC AUC**	**PR AUC**	**Precision**	**Recall**	F_1
Logistic Regression	0.83	0.85	0.92	0.95	0.82	0.88
LDA	0.82	0.87	0.94	0.94	0.81	0.87
SVM (linear)	0.95	0.99	1	0.96	0.98	0.97
SVM (RBF)	0.92	0.99	1	0.92	0.98	0.95
Greedy Forward	0.87	0.93	0.98	0.95	0.88	0.91
Greedy Backward	0.82	0.85	0.92	0.93	0.82	0.87
**Random Forest**	0.94	0.99	1	0.94	1	0.97

Table S1: **Benchmark of classification methods under identical cross-validation.** All models were evaluated with stratified 5-fold *outer* CV; where applicable, hyperparameters were selected by stratified 5-fold *inner* CV (training fold only). Values are outer-fold means (rounded to two significant figures). Positive class probabilities were used to compute ROC AUC and PR AUC; thresholded metrics used a 0.5 cutoff.

S2 Additional detail on biomarker descriptions

Immunological Feature (63 total)	Brief Description
IgG Serum Spike	Systemic IgG antibodies in blood targeting the SARS-CoV-2 spike protein; indicates systemic humoral immunity.
IgG Serum RBD	IgG in blood targeting the receptor-binding domain (RBD) of spike; involved in virus neutralization.
IgG Saliva Spike	Mucosal IgG in saliva targeting spike; reflects mucosal immune response.
IgG Saliva RBD	Mucosal IgG in saliva targeting the RBD; may correlate with protection at mucosal surfaces.
IgA Saliva Spike	Secretory IgA in saliva targeting spike; key for mucosal immunity, especially prevention of initial infection.
IgA Saliva RBD	Secretory IgA in saliva targeting the RBD; key for mucosal immunity, especially prevention of initial infection.
IFNγ (Interferon γ)	Cytokine produced by activated T cells (especially Th1); critical for antiviral immunity and macrophage activation.
IL-2 (Interleukin-2)	Cytokine produced by activated T cells that promotes T-cell proliferation and survival; important for immune memory.
Dual (IL-2 + IFNγ)	T cells simultaneously producing IL-2 and IFNγ; polyfunctional responses associated with robust immunity.
Neutralization	Functional antibody assay measuring the ability of antibodies to prevent viral infection of host cells.
ACE2 Displacement	Surrogate for neutralization; measures the ability of antibodies to block viral RBD binding to the ACE2 receptor.

Table S2: Overview of the immunological feature families used in this study. Individual measurements (63 total) fall into the categories listed; detailed assay descriptions and per-feature metadata are provided in Methods/Supplement.

S3 PWH viral load metadata

Table S3: Patient metadata summary.

Number	Patient ID	HIV diagnosis	Years VL suppressed
24	CIRC 0054	1988	LTNP
25	OM 215	1993	28
26	OM 5085	1985	20
27	OM 5076	1989	21
28	CIRC 0022	1995	23
29	OM 5056	1987	24
30	CIRC 0116	2000	19
31	OM 5030	1985	20
32	CIRC 0050	1989	23
33	OM 5135	1988	17
34	OM 5208	1989	17
35	CIRC 0120	1989	16
36	CIRC 0313	1984	20
37	OM 5051	1990	23
38	CIRC 0041	1999	22
39	OM 5202	2008	12
40	CIRC 0280	1992	11
41	OM 5055	2002	25
42	OM 5013	1989	25
43	CIRC 0066	1989	24
44	OM 5128	2002	17
45	OM 5130	1990	16

Continued on next page

Continued from previous page

Number	Patient ID	HIV diagnosis	Years VL suppressed
46	OM 5265	2011	10
47	OM 5232	2000	21
48	OM 5368	2015	5
49	OM 5407	1992	7
50	OM 5248	1998	20
51	OM 5365	1990	18
52	OM 5200	1990	24
53	OM 5225	1999	21
54	CIRC 0196	2007	8
55	CIRC 0281	1997	13
56	CIRC 0146	1990	15
57	CIRC 0113	1993	19
58	CIRC 0188	1996	20
59	CIRC 0323	1990	19
60	OM 5213	1993	20
61	CIRC 0273	2000	16
62	CIRC 0302	2000	17
63	CIRC 0164	2006	11
64	OM 5122	2007	13
65	OM 5409	2007	12
66	CIRC 0028	1991	30
67	CIRC 0324	1987	16
68	OM 5400	1989	15
69	OM 5005	1987	15
70	CIRC 0216	2000	17

Continued on next page

Continued from previous page

Number	Patient ID	HIV diagnosis	Years VL suppressed
71	OM 5016	1992	14
72	CIRC 0322	1990	14
73	CIRC 0266	1988	16
74	CIRC 0060	1994	24
75	OM 5168	1985	20
76	OM 5156	1985	14
77	CIRC 0270	1985	22
78	CIRC 0274	2000	20
79	OM 5131	1982	15
80	CIRC 0319	1990	30
81	OM 5244	1986	20
82	CIRC 0058	1993	23
83	OM 5226	2005	16
84	CIRC 0174	1994	20
85	CIRC 0036	1991	30
86	CIRC 0308	1988	19
87	OM 5094	1985	NA
88	OM 5019	2005	NA
89	OM 5004	1981	NA
90	CIRC 0106	1990	NA
91	OM 5211	2000	NA

S4 RF classification probabilities

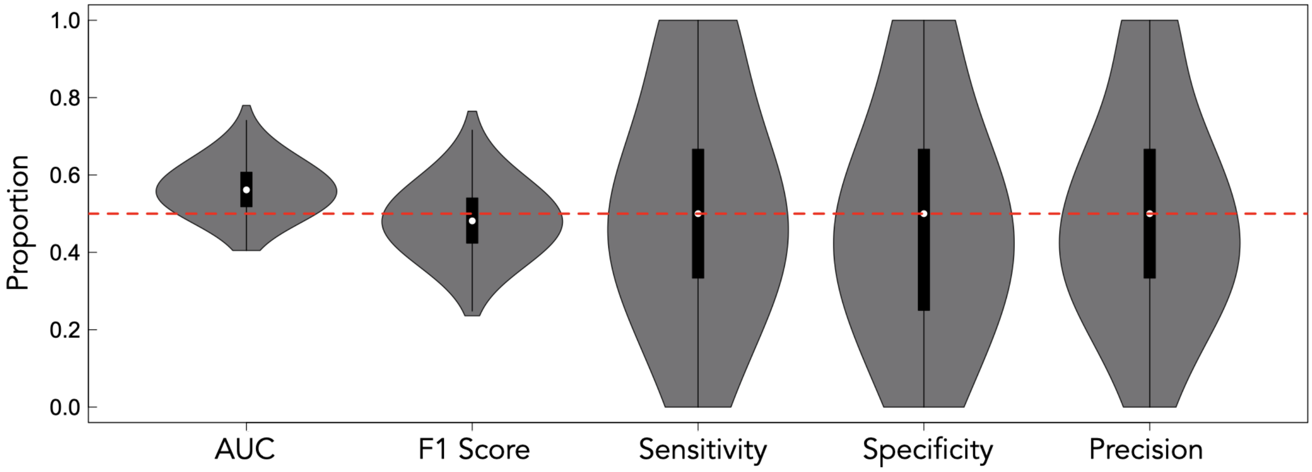

Figure S1: **Model performance with randomized labels using all 63 features.** RF was carried out similarly to as described in the methods section with K-fold CV, however, the labels were randomized. Provided are the AUC, F1 Score, Sensitivity, Specificity, and Precision, respectively, which can be compared to the true model performance metrics provided in Fig 1E. A red dashed line at 0.5 is shown for comparison

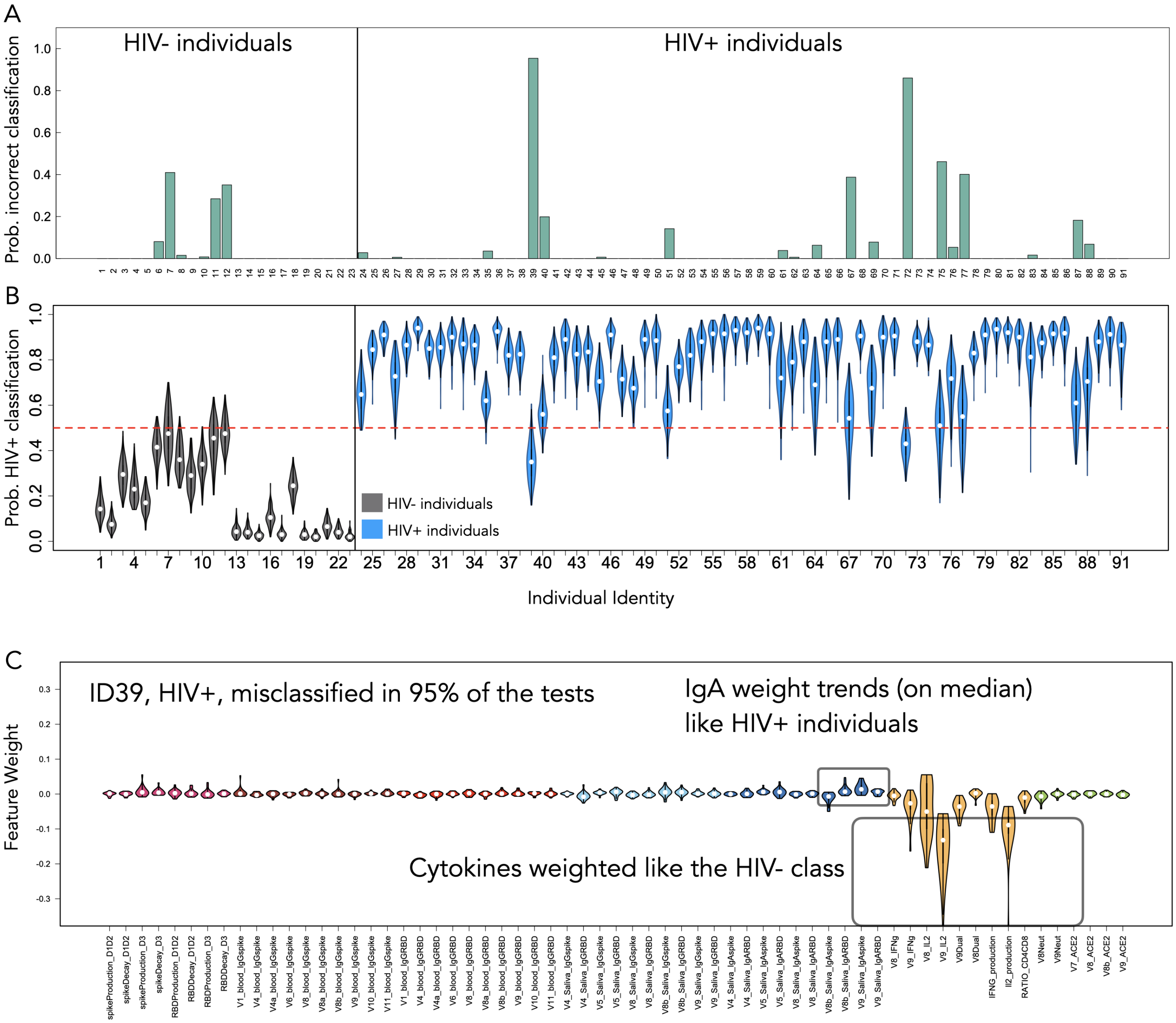

Figure S2: **Classification probabilities across all folds for HIV- and HIV+ individuals and misclassified HIV+ feature weight signature.** A) Probability that the RF model will incorrectly classify the individual, for all individuals in the full-feature RF models. B) Violin plots for the distributions of HIV+ classifications for all individuals. C) Example feature weight distributions for ID39, who tends to be misclassified as HIV- when they are known to be HIV+.

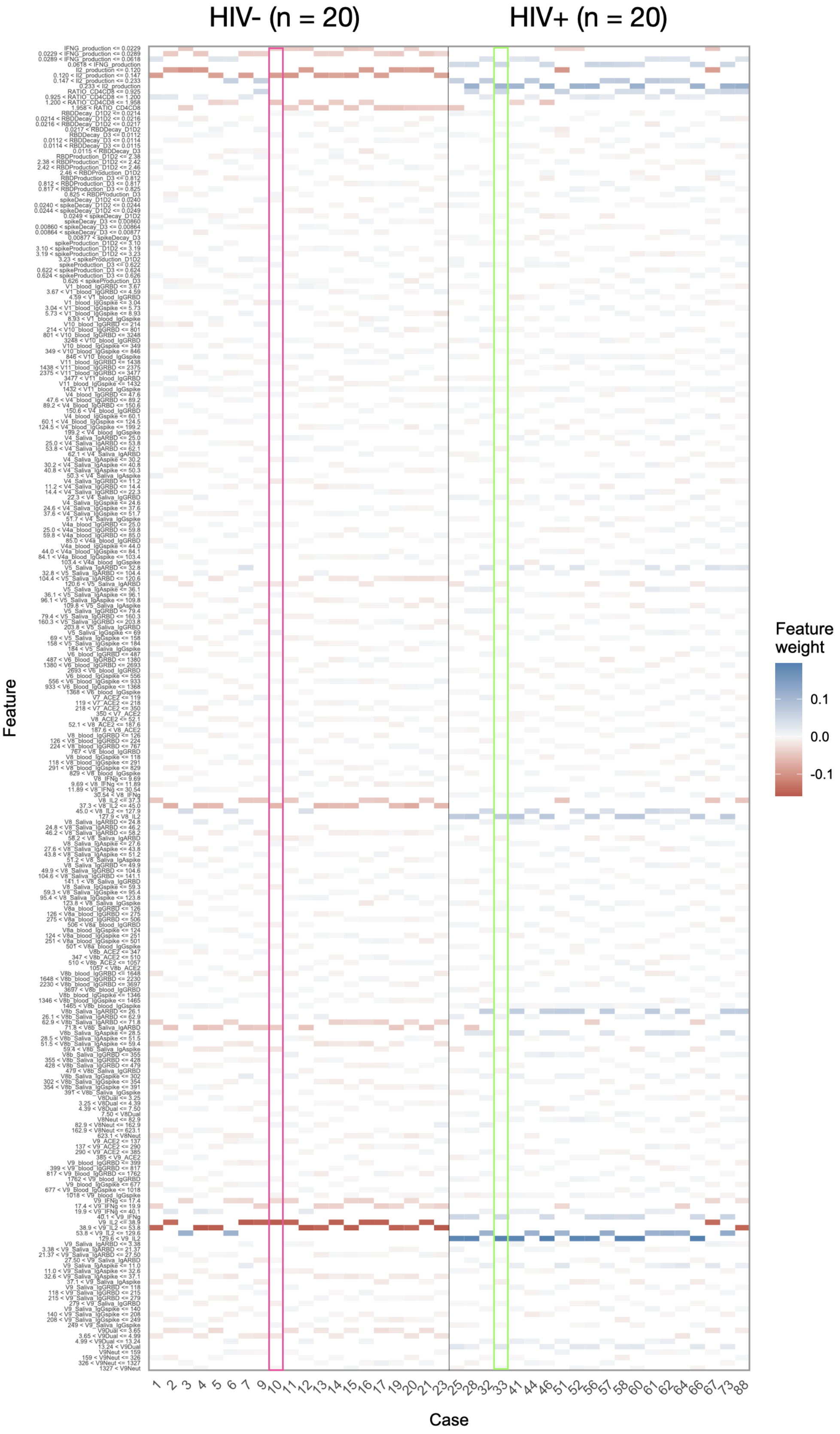

Figure S3: **RF training landscape example.** An example of the RF training landscape for a single RF model, IDs 10 and 33 are randomly highlighted, with their corresponding individual feature distributions shown in Fig. S4. This RF model produces an AUC of 1.0.

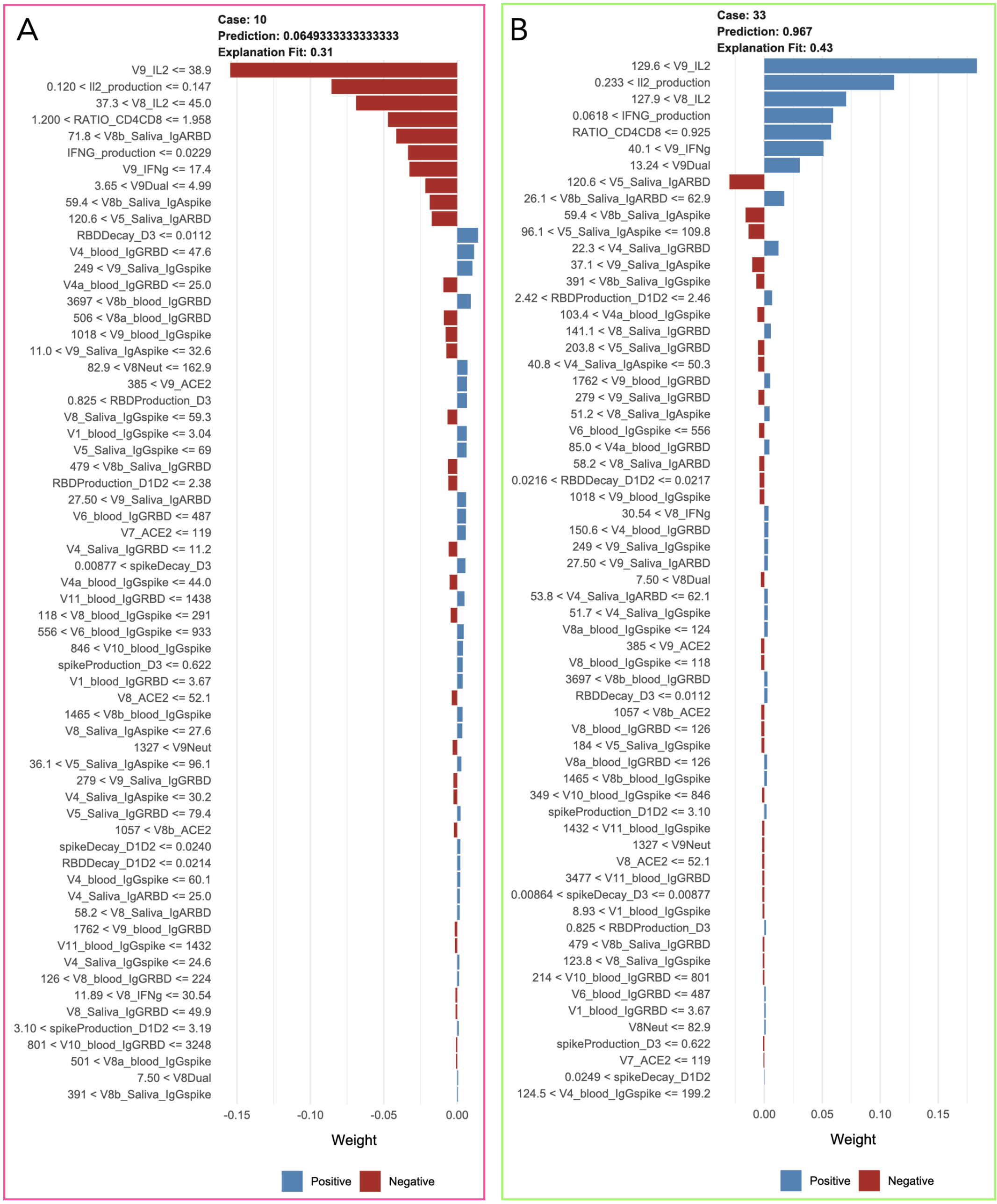

Figure S4: **Individual feature weights.** Individual feature weights for IDs 10 and ID 33 for a single RF model trained on all 64 features from the model landscape shown in Fig. S3.

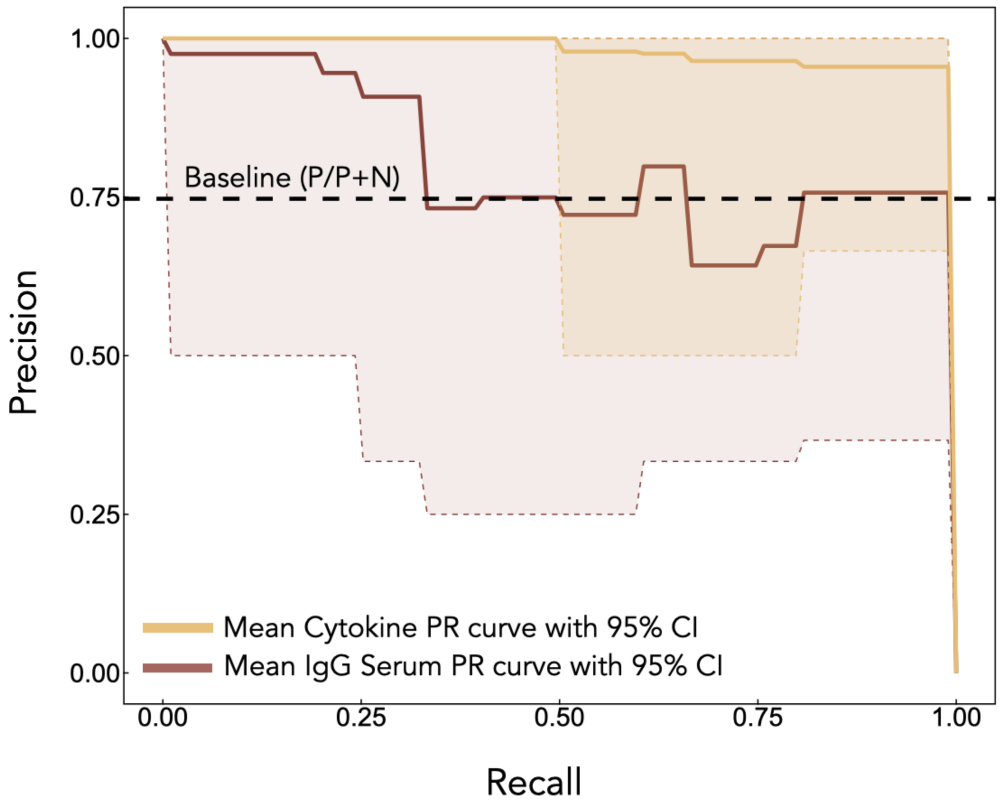

Figure S5: **Mean PR curves for RF models trained on just the cytokine features (yellow) and serum data (red).** Shaded regions represent 95% confidence intervals. The baseline (dashed line) is calculated by computing the ratio $P/(P+N)$ which is the ratio of positives and negatives in the full data set (not the downsampled balanced ratio of 1:1 used for training, but the representative ratio used for testing). In accordance with intuition gained from the mean ROC curves (Fig. 3E), the cytokine features result in a near-perfect classifier while serum features result in approximately baseline (random) performance.

S4.1 Synthetic Data Analysis

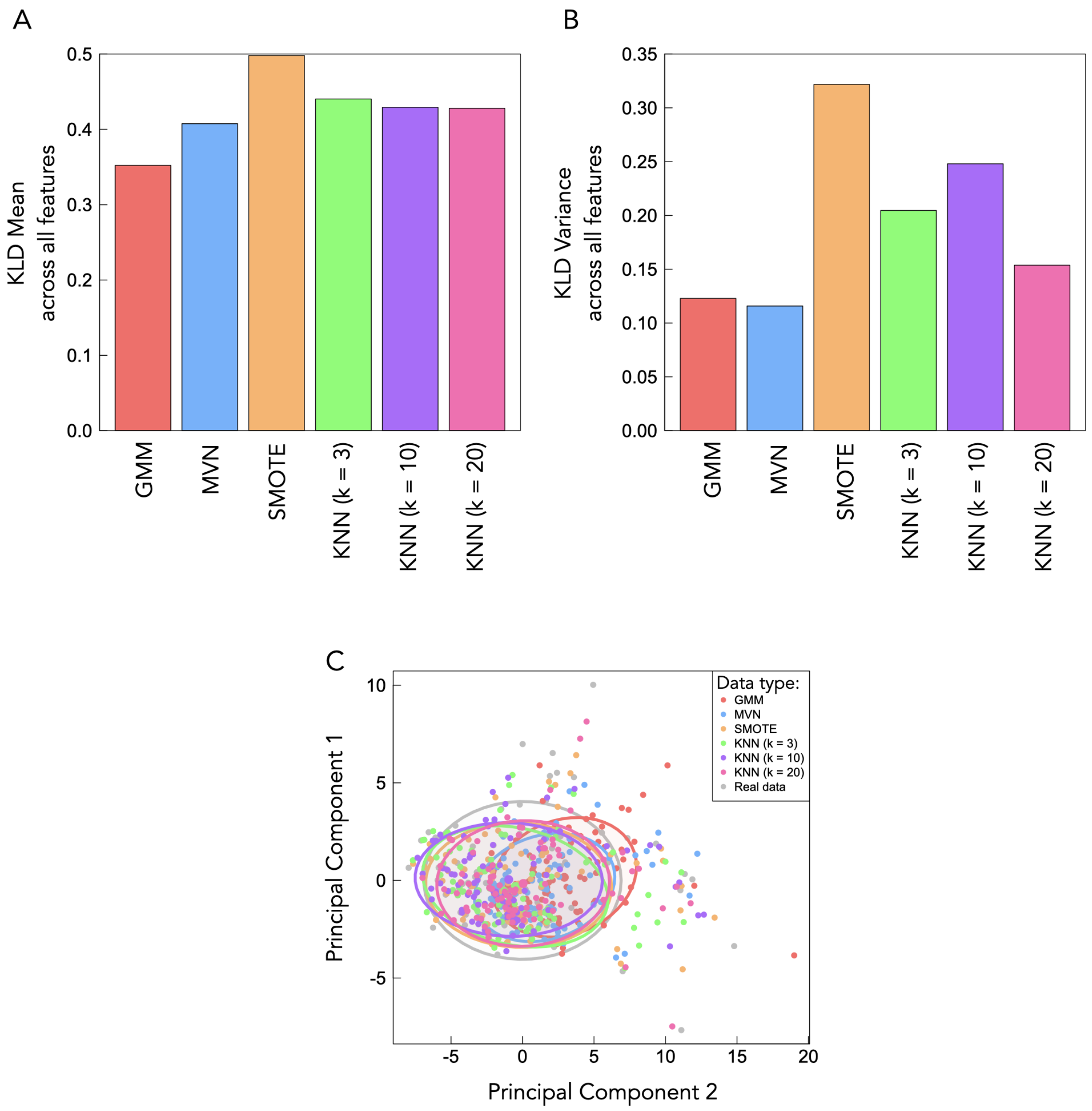

Figure S6: **Synthetic data KLD and PCA analysis.** A) Mean KLD is computed across all features between each respective synthetic data set and the real data. B) The variance in KLD is computed across all features between each respective synthetic data set and the real data. C) PCA analysis is displayed whereby the principal components from the real data are projected onto each respective principal components of the synthetic data. Ellipses are first standard deviation. Here, all synthetic data approaches are reasonably approximate the actual data and no meaningful cluster separation is found.

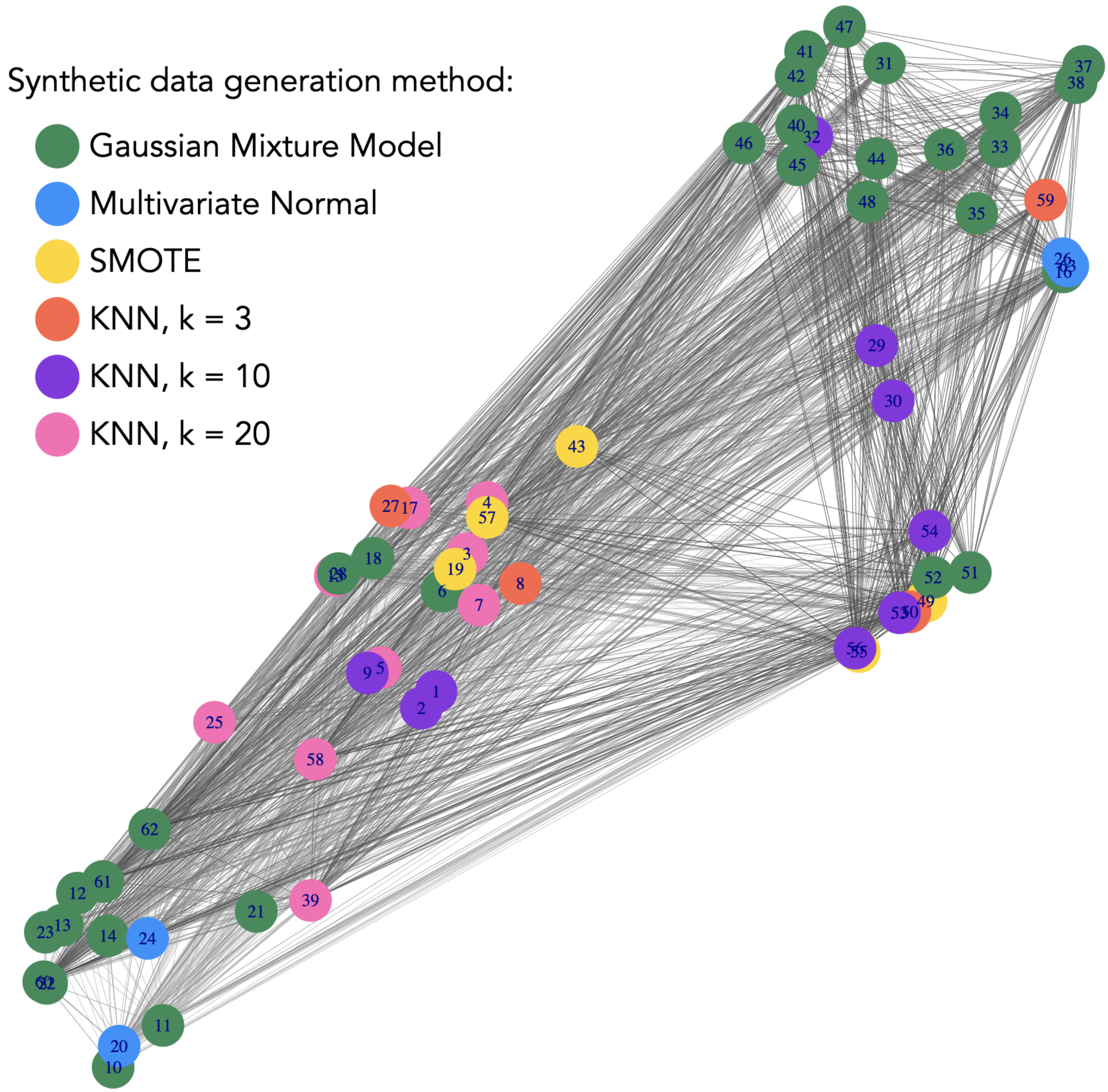

Figure S7: t-SNE plot shown with the same layout as in the maintext, however, colours here correspond to the synthetic data feature method that minimized the KLD for that specific feature.

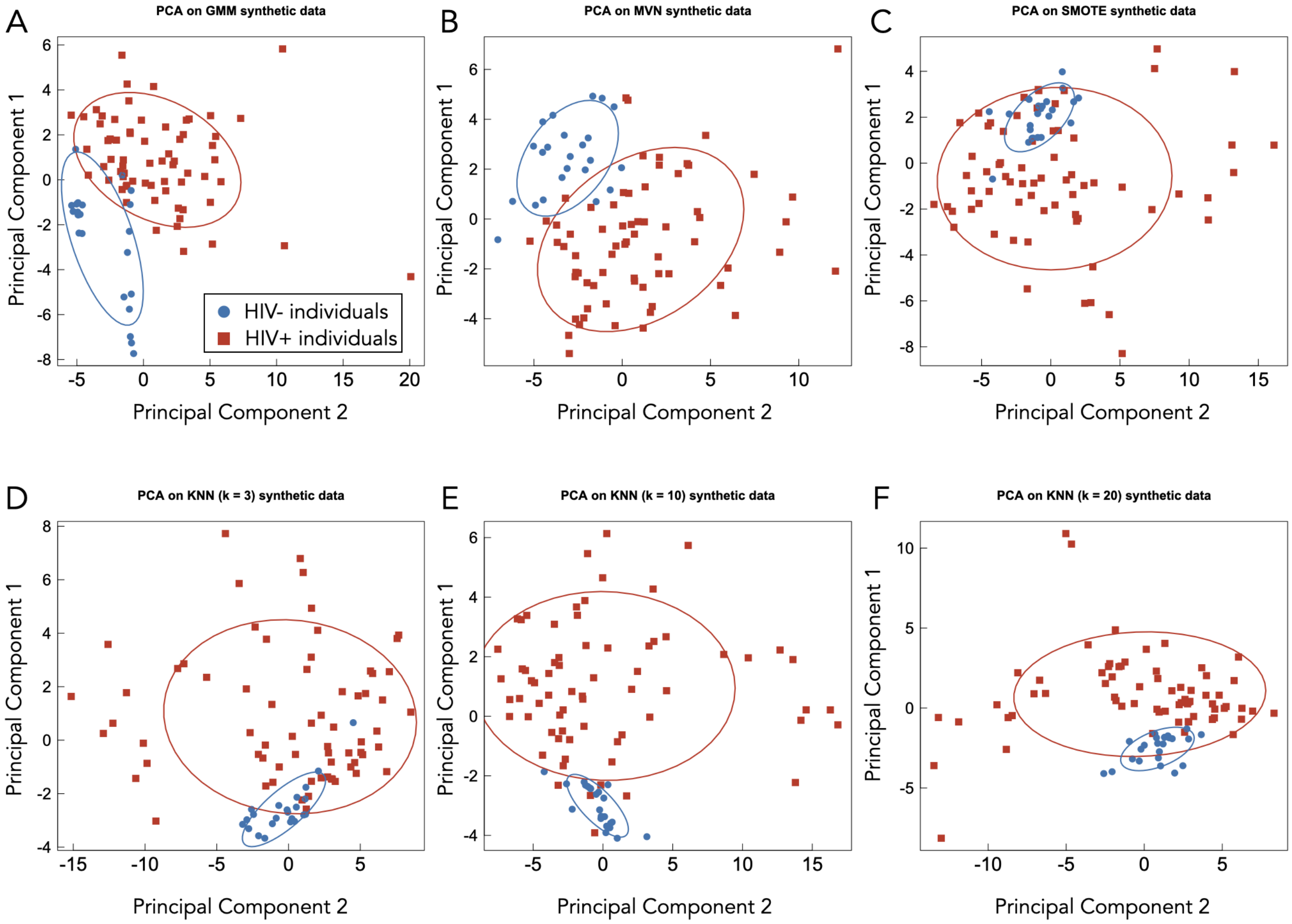

Figure S8: **PCA on synthetically generated data**. Panels A-F display the results of PCA performed on the synthetically generated data. Panels are ordered by GMM, MVN, SMOTE, KNN (k = 3), KNN (k = 10), and KNN (k = 20), for panels A, B, C, D, E, and F, respectively. SMOTE, despite having the highest mean KLD, is the only technique whereby the control class subclusters within the first standard deviation ellipse of the target class, which is similar structural behaviour observed by the actual data (Fig.1C). SMOTE is the only supervised method used to generate synthetic data in this work.

28 ## S5 Imputation analysis

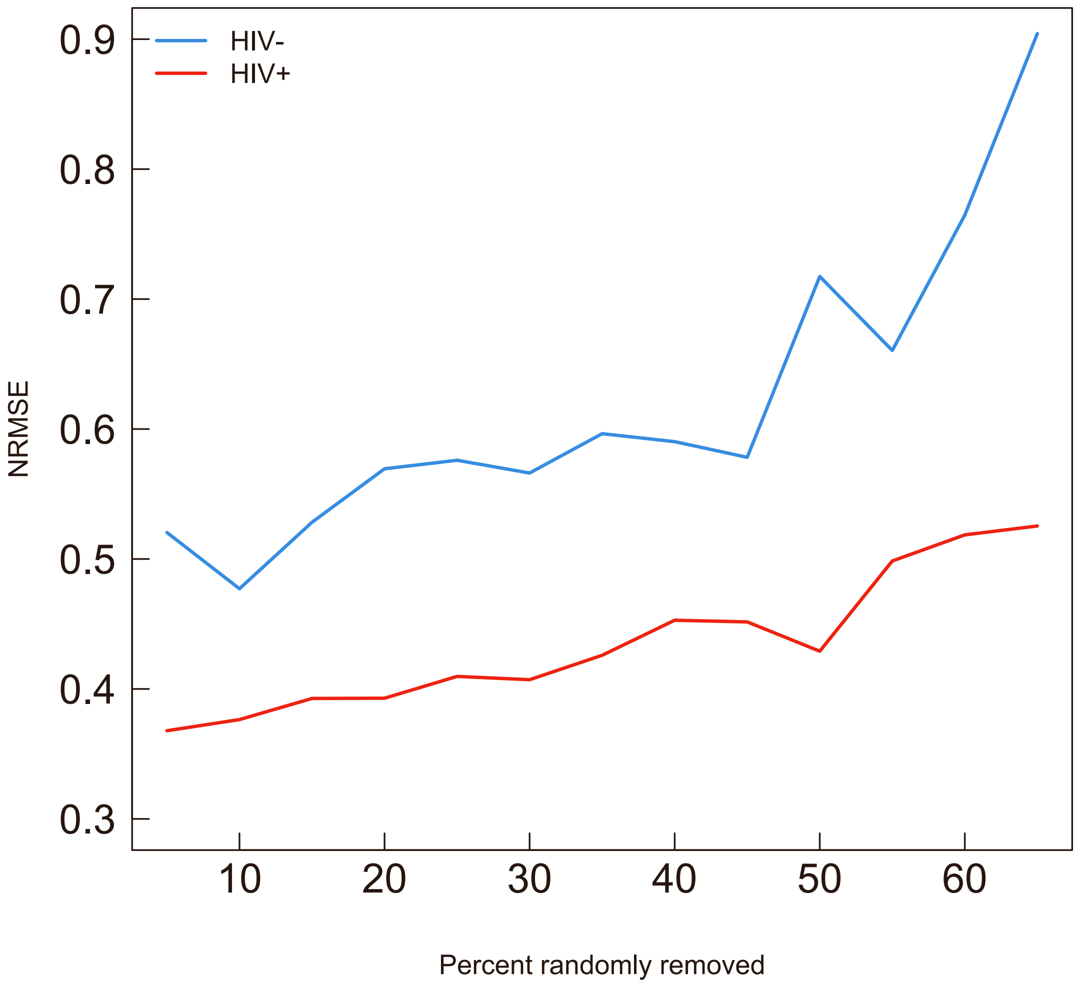

Figure S9: **Normalized Root Mean Squared Error (NRMSE) of imputed features as a function of increasing missingness.** NRMSE was computed separately for HIV-negative (blue) and HIV-positive (red) groups, with each point representing the average NRMSE across all features at a given level of missing data. As expected, NRMSE increases gradually as the proportion of missing data increases, reflecting the increasing uncertainty in imputed values. However, the shallow slope suggests that the imputation method remains robust across varying levels of missingness, preserving data integrity even at higher rates of removal.

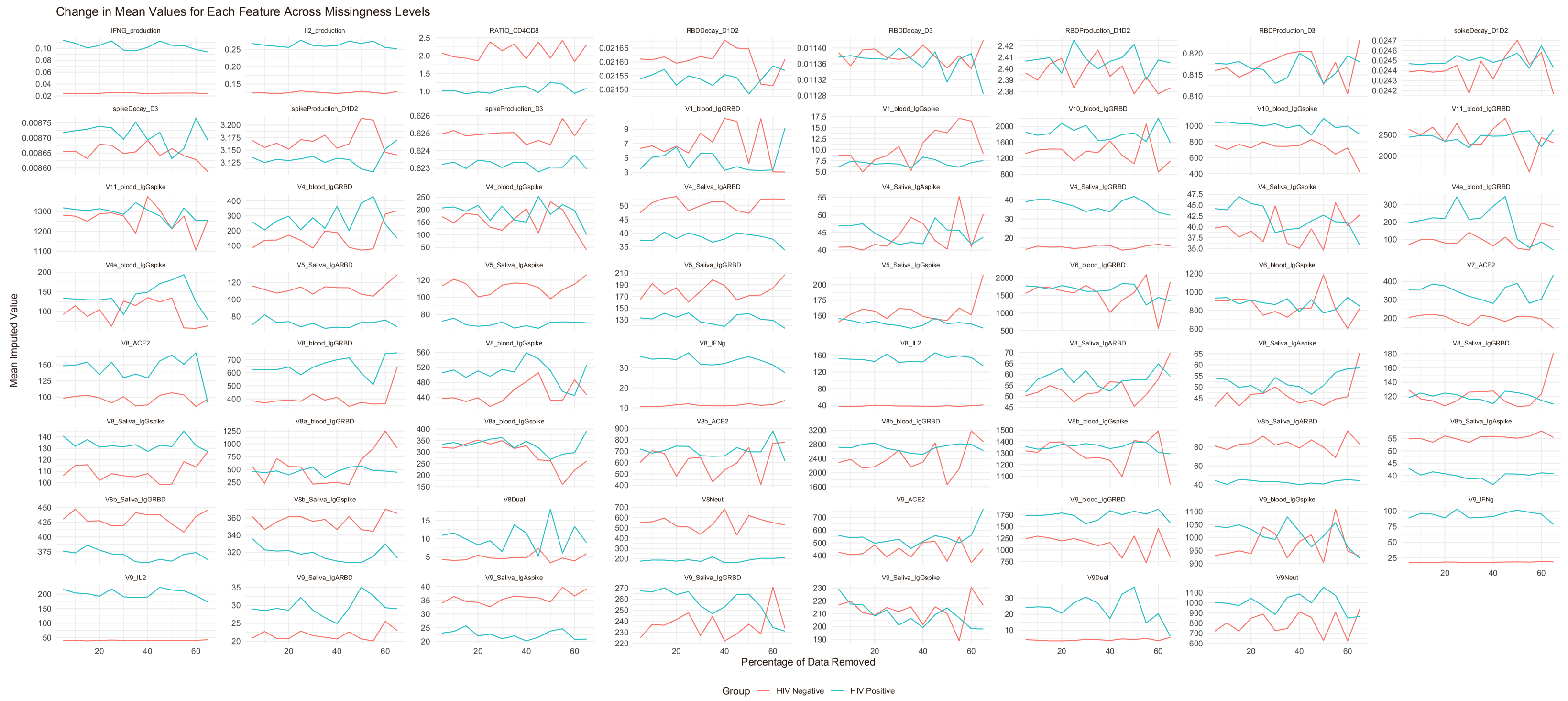

Figure S10: **Mean values of the imputed features remains stable across increasing levels of missingness.** Each panel represents a single feature, with the x-axis indicating the percentage of data removed prior to imputation, and the y-axis representing the mean of the imputed values. Imputation was performed separately for HIV-negative (blue) and HIV-positive (red) groups as described in the methods section. The relative stability of these metrics suggests that the imputation approach preserves the underlying data structure, even as missingness increases.

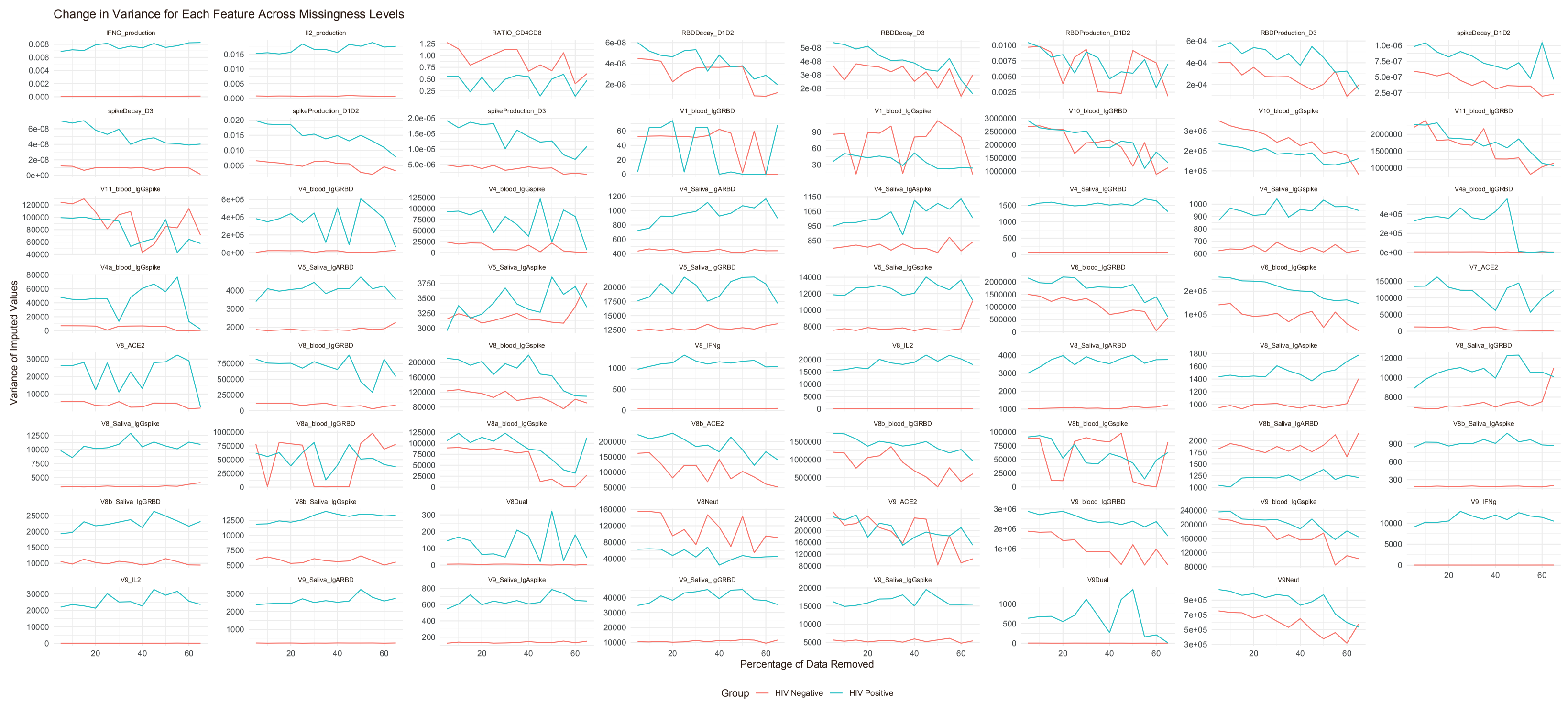

Figure S11: **Variance of the imputed features remains stable across increasing levels of missingness**. Each panel represents a single feature, with the x-axis indicating the percentage of data removed prior to imputation, and the y-axis representing the mean of the imputed values. Imputation was performed separately for HIV-negative (blue) and HIV-positive (red) groups as described in the methods section. The relative stability of these metrics suggests that the imputation approach preserves the underlying data structure, even as missingness increases.

S6 UMAP to compliment tSNE

As validation to confirm the stability and biological relevance of the tSNE clusters presented in Figure 2, here we look at feature clustering behvaiour using UMAP. We use the umap function in the R library uwot and set the effective neighbors to the same as the perplexity value used in the tSNE analysis.

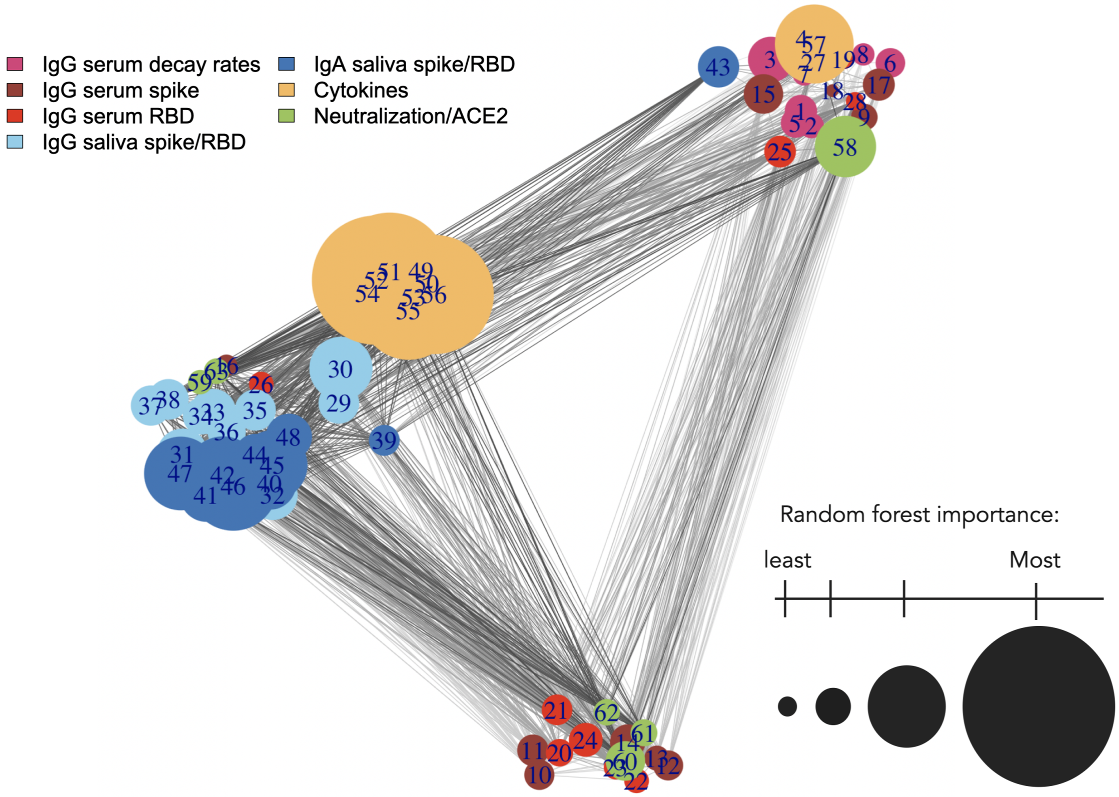

Figure S12: **UMAP of the feature correlation network.** UMAP embedding of the feature–feature correlation network. Nodes (1–63) are immune features; edges connect pairs with Spearman associations across the 91 participants with $p < 0.05$. The layout uses UMAP with $n_{\text{neighbors}} \approx (63 - 1)/3 = 20$, `min_dist`$= 0.1$, and Euclidean metric. Node colours denote assay families (magenta: IgG serum decay rates; red: IgG serum spike; maroon: IgG serum RBD; light blue: IgG saliva spike/RBD; dark blue: IgA saliva spike/RBD; orange: Cytokines; green: Neutralization/ACE2). Node size is proportional to random-forest feature importance.
